# Mapping distribution of cysts of recent dinoflagellate and *Cochlodinium polykrikoides* using next-generation sequencing and morphological approaches in South Sea, Korea

**DOI:** 10.1038/s41598-018-25345-4

**Published:** 2018-05-03

**Authors:** Seung Won Jung, Donhyug Kang, Hyun-Jung Kim, Hyeon Ho Shin, Joon Sang Park, So Yun Park, Taek-Kyun Lee

**Affiliations:** 1 Library of Marine Samples, Korea Institute of Ocean Science & Technology, Geoje, 53201 Republic of Korea; 2 Maritime Security Research Center, Korea Institute of Ocean Science & Technology, Busan, 49111 Republic of Korea; 3 Marine Ecosystem and Biological Research Center, Korea Institute of Ocean Science & Technology, Busan, 49111 Republic of Korea; 4 South Sea Environment Research Center, Korea Institute of Ocean Science & Technology, Geoje, 53201 Republic of Korea

## Abstract

The total dinoflagellate cyst community and the cysts of *Cochlodinium polykrikoides* in the surface sediments of South Sea (Tongyeong coast), South Korea, were analysed using next-generation sequencing (NGS) and morphological approaches. Dinoflagellate cysts can be highly abundant (111–4,087 cysts g^−1^ dry weight) and have diverse species composition. A total of 35 taxa of dinoflagellate cysts representing 16 genera, 21 species (including four unconfirmed species), and 14 complex species were identified by NGS analysis. Cysts of *Scrippsiella* spp (mostly *Scrippsiella trochoidea*) were the most dominant and *Polykrikos schwartzii*, *Pentapharsodinium dalei*, *Ensiculifera carinata*, and *Alexandrium catenella/tamarense* were common. Thus, a combination of NGS and morphological analysis is effective for studying the cyst communities present in a given environment. Although *C. polykrikoides* developed massive blooms during 2013–2014, microscopy revealed low density of their cysts, whereas no cysts were detected by NGS. However, the vegetative *C. polykrikoides* not appeared during 2015–2017 in spite of the observation of *C. polykrikoides* cysts. This suggests that the *C. polykrikoides* blooms were not due to development of their cysts but to other factors such as currents transporting them to a marine environment suitable for their growth.

## Introduction

Dinoflagellates are one of the most diverse and abundant groups of marine plankton. Some dinoflagellates are known to cause outbreaks of harmful algal blooms in coastal waters around the world, and can be extremely toxic, leading to significant damage of local aquaculture and wildlife. The life stages of dinoflagellates form an important component of their ecology and biogeography. Around 200 of the approximately 2000 existing species of marine dinoflagellates are known to form resting cysts as part of their life history^1^. Cysts are typically preserved in sediments, thereby providing an integrated record over time of the presence of cyst-producing dinoflagellates. Functions attributed to cysts include species dispersal, survival under unfavourable conditions, and bloom initialization^2–4^. Cyst surveys give information on history of harmful species in a given area, thereby providing an indication of the potential for future blooms, and it may reveal species rarely observed in the plankton, owing to rare, short-lived, or vegetative stages that are difficult to identify^5^.

In South Korea, *Cochlodinium polykrikoides* has been one of the most frequently occurring harmful red tide species responsible for fish kills since 1995^6^. In particular, the Tongyeong coastal area, located in the South Sea of South Korea, is one of the most severely bloomed areas of *C. polykrikoides*^7^. Although, *C. polykrikoides* blooms frequently developed in many coastal countries during the last decade, cysts of *C. polykrikoides* are still understudied^8–10^. Our previous study reported the cyst morphology of *C. polykrikoides* in the natural surface sediment samples^9^, but the mechanisms of *C. polykrikoides* bloom formation by germination of cysts are mostly unknown.

Advances in DNA sequencing technology and bioinformatics have significant potential to strengthen biological monitoring in the ocean. The challenge of modelling and predicting ecological changes requires the linking of taxonomic data to the functional roles of individual microbial groups in biogeochemical cycles^11^. In other words, the challenge is not simply a matter of discovering ‘What is there’, but also ‘Why it is there?’ In this context, a major issue relates to how dinoflagellate cysts are temporally distributed and whether they follow any trends in their distribution.

To this end, we investigated the diversity and distribution of cysts in dinoflagellate communities and *C. polykrikoides* by focusing on 18S rDNA using next-generation sequencing (NGS) tool and morphological observation in samples harvested from the South Sea (Tongyeong coasts) during the red-tide and the non-red-tide season in South Korea.

## Results

### Dinoflagellate cyst composition based on next-generation sequencing analysis

**Figure 1:**
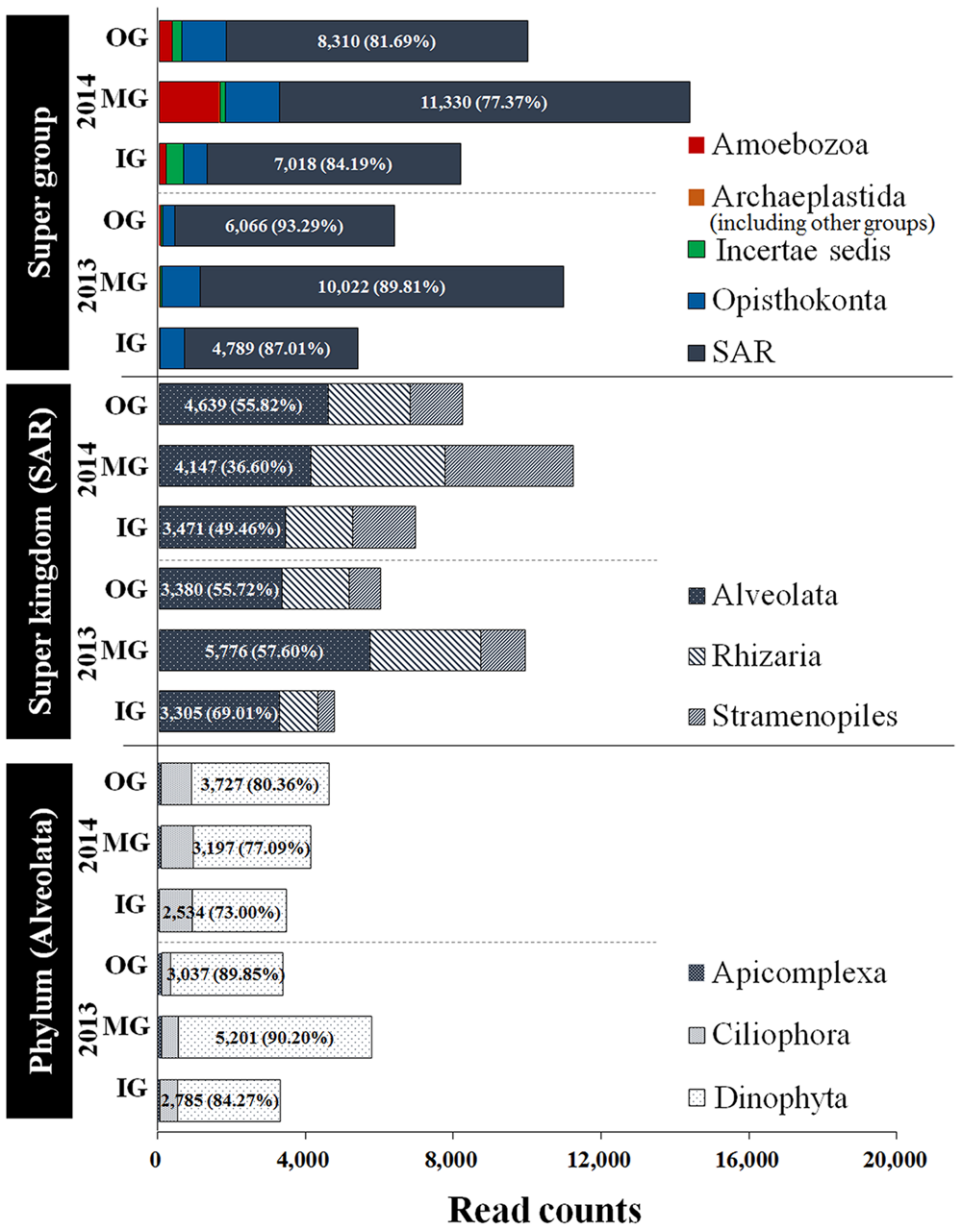
Taxonomic distribution of super group, super kingdom, and phylum levels revealed by next-generation sequencing analysis in surface sediments of Tongyeong coast (South Sea) of South Korea during December 2013 and September 2014. IG, inner group; MG, middle group; OG, outer group; SAR, Stramenopiles-Alveolata-Rhizaria super kingdom.

The NGS results obtained for the eukaryotic organisms in surface sediments of Tongyeong coasts are summarized in Table 1. The number of OTUs varied among samples. The rarefaction curves (Supplementary Fig. S1), which plot the OTUs generated by the NGS runs against the number of reads, appeared to be nearing the asymptotic values. The amplicons generated 31,575–81,818 read counts from the six sequenced samples: The average read count in 2014 (59,894) were higher than in 2013 (42,383). In addition, the number of OTUs and alpha diversity (Chao1 richness, Shannon diversity, and Simpson evenness) showed a similar trend with changes in read counts (Table 1). Read counts, number of OTUs, and alpha diversity in the MG group in 2013 were higher than those in the IG and OG. However, the data of 2014 showed that the number of OTUs and alpha diversity of the OG were higher than in the other groups. Considering that eukaryotic organisms are present in sediments, the main group identified in the NGS data (97% cut off of read counts) was the Stramenopiles-Alveolata-Rhizaria (SAR) super group, with average proportion of 90.04% and 81.08% in 2013 and 2014, respectively (Fig. 1). Other groups, such as Opisthokonta, Archaeplastida, Amoebozoa, and incertae sedis were minor groups. In the SAR super group, Alveolata was the most abundant super kingdom with a average proportion of 60.78% in 2013 and 47.29% in 2014, followed by Rhizaria and Stramenopiles. Three diverse groups of unicellular eukaryotes were consisted of the Alveolata. Among these phyla, Dinoflagellata occupied an extremely high average proportion of 88.11% in 2013 and 76.82% in 2014 (Fig. 1).

**Table 1:** Number of read counts and operational taxonomic units (OTUs) obtained from next-generation sequencing analysis and the alpha-diversity indices in this study.

Sampling date	group	Total bases	Read Count (Cut off: 97%)	OTUs	Chao1 richness	Shannon diversity	Simpson evenness	Remark
December 2013	IG	13,141,208	31,575(5,504)	175	190	4.82	0.87	Non red-tide
	MG	25,586,779	61,246(11,159)	227	242	5.28	0.91	
	OG	14,315,268	34,327(6,502)	208	217	5.24	0.90	
September 2014	IG	18,935,579	45,435(8,336)	256	271	5.61	0.94	Red-tide
	MG	34,212,216	81,818(14,646)	264	287	4.91	0.92	
	OG	21,940,670	52,428(10,173)	273	292	5.42	0.92	

IG, MG, and OG indicate inner group, middle group, and outer groups, respectively, of sample sites in Tongyeong coast (South Sea) of South Korea during December 2013 and September 2014.

**Figure 2:**
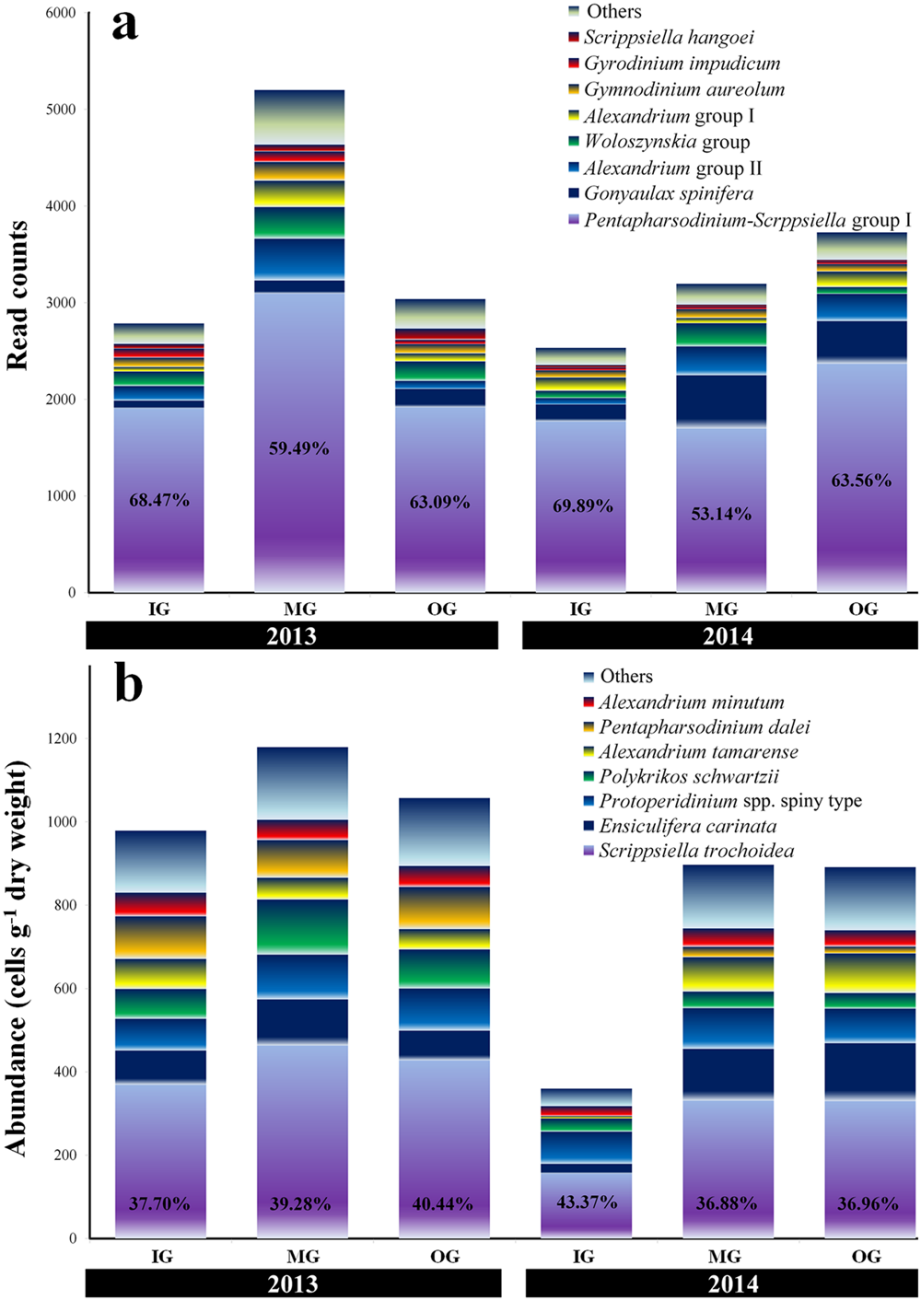
Common species (or genus) composition-inferred assemblages of dinoflagellate cysts based on next-generation sequencing (**a**) and morphological data (**b**) in surface sediments of Tongyeong coast (South Sea) of South Korea during December 2013 and September 2014. IG, inner group; MG, middle group; OG, outer group.

The composition of the dinoflagellate cyst assemblages is shown in Fig. 2a (97% cut-off of read counts) and Supplementary Tables S1 and S2. A total of 35 taxa (or complex species) of cysts representing 16 genera, 21 species (including four unconfirmed species), and 14 groups (complex species) were identified from all the samples. The *Pentapharsodinium*-*Scrippsiella* complex, which included *Pentapharsodinium tyrrhenicum*, *P. dalei*, *Scrippsiella trochoidea*, *S. sweeneyae*, and *S. erinaceus*, constituted the most predominant taxa with average proportions of 63.68% in 2013 and 62.20% in 2014. *Gonyaulax spinifera* (3.94% in 2013 and 11.99% in 2014), *Alexandrium* complex species I, including *Alexandrium pacificum*, *A. catenella/tamarense*, and *A. fundyense* (5.49% in 2013 and 6.50% in 2014), *Woloszynskia* complex species, including *Woloszynskia cincta* and *W. halophila* (6.09% in 2013 and 4.17% in 2014), *Alexandrium* complex species II, including *A. minutum*, *A. insuetum*, *A. tamutum*, and *A. lusitanicum* (3.23% in 2013 and 3.74% in 2014), *Gymnodinium aureolum* (3.47% in 2013 and 2.65% in 2014), *Gyrodinium impudicum* (2.29% in 2013 and 0.99% in 2014), and *S. hangoei* (2.31% in 2013 and 0.62% in 2014) were the next most abundant species and represent more than 1% of the total read counts of OTUs. These major taxa account for more than 90% of the total read counts of cysts of dinoflagellates. In particular, *G. spinifera* was present in higher proportion in 2014 compared to 2013.

### Dinoflagellate cyst composition based on microscopic analysis

**Figure 3:**
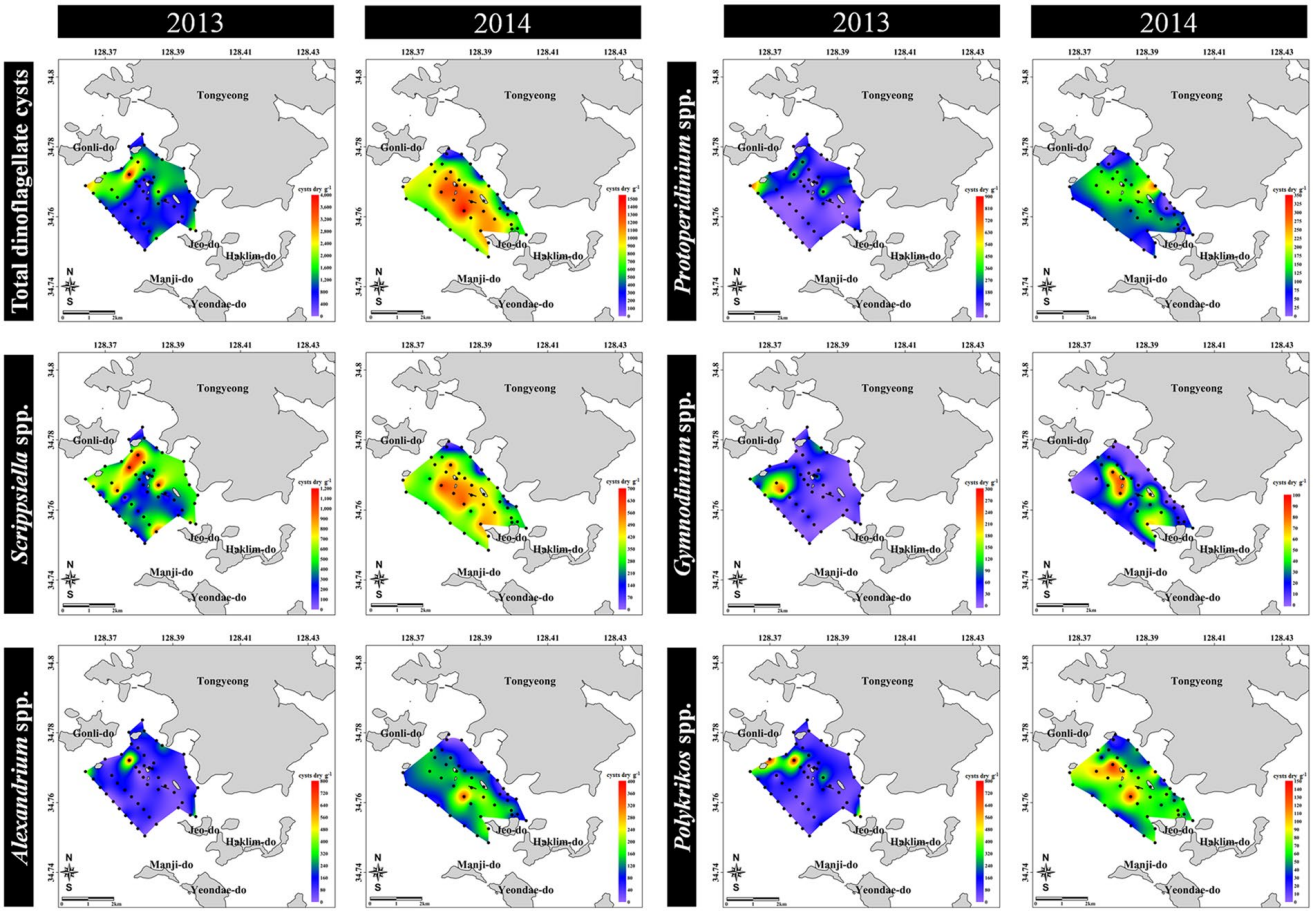
Distribution of total and common dinoflagellate species revealed by morphological analysis in surface sediments of Tongyeong coast (South Sea) of South Korea during December 2013 (50 sampling sites) and September 2014 (44 sampling site). *Scrippsiella* spp., including *S. trochoidea*, *S. tridida*, *S. precaria*, *S. crystalline*, *S. rotunda*, and *S. operosa*; *Alexandrium* spp., including *A. catenella/tamarense* and *A. minutum*; Protoperidinium spp., including *P*. *latissimum*, *P*. *leonis*, *P*. *minutun*, *P. americanum*, and *Protoperidinium* spp. spiny type; *Gymnodinium* spp., including *G. catenatum* and *Gymnodinium* spp.; *Polykrikos* spp., including *P. schwartzii* and *P. kofoidii*. All maps were generated with Surfer v12.2.705 (http://www.goldensoftware.com/products/surfer).

In microscopic observations, the total abundance of dinoflagellate cysts at the sampling sites varied between 253–4,087 cysts g^−1^ dry weight (average = 1,072 cysts g^−1^ dry weight) in 2013 and 111–1,532 cysts g^−1^ dry weight (average = 716 cysts g^−1^ dry weight) in 2014 (Fig. 3). Thus, the dinoflagellate cysts in 2013 showed higher abundance than in 2014 (Fig. 3). In particular, the abundance of cysts of the MG in both 2013 and 2014 was higher than in the other groups. Thus, our samples of the dinoflagellate cysts in the surface sediments of the Tongyeong coast revealed high abundance and diverse species composition, compared to the abundance and composition in many coastal countries worldwide (Supplementary Table S3). In addition, abundances recorded in this study were higher than those reported previously for other coastal areas of South Korea (Supplementary Table 3). The composition of the dinoflagellate cysts is shown in Fig. 2b and Supplementary Table S4. A total of 25 taxa of cysts representing 13 genera and 25 species (including five unconfirmed species) were identified in 2013, and 27 taxa representing 13 genera, 27 species (including five unidentified cysts), and one unidentified taxon in 2014. The cysts of *Scrippsiella* spp (mostly *Scrippsiella trochoidea*) were the most predominant at average proportions of 40.72% (27.93% for *S. trochoidea*) in 2013 and 43.55% (21.05% for *S. trochoidea*) in 2014. Moreover, the predominant cysts (>5% in total cyst abundances) in 2013 were of *Polykrikos schwartzii* (9.65%), *Protoperidinium* sp. (spiny form, 9.01%), *Pentapharsodinium dalei* (8.82%), *Ensiculifera carinata* (8.24%), *Phaeopolykrikos hartmanii* (5.23%), and *Alexandrium catenella/tamarense* (5.07%) and the predominant cysts in 2014 were of *Ensiculifera carinata* (13.77%), *Protoperidinium* sp (spiny form, 11.37%), and *Alexandrium catenella/tamarense* (8.94%). Thus, the proportion of total common cysts (average cyst abundances > 5% in 2013 and 2014) were 77.92 and 77.63%, respectively (Fig. 3 and Supplementary Fig. S2).

### Similarity in dinoflagellate cyst distribution based on the NGS and microscopic analysis

**Figure 4:**
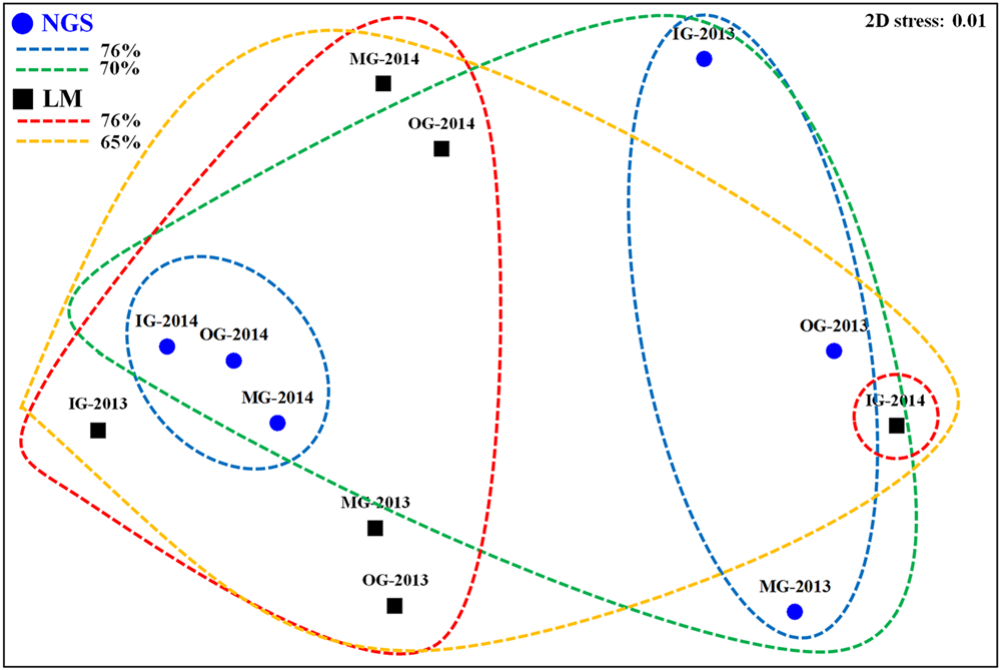
Non-metric multidimensional scaling plot based on next-generation sequencing (NGS as blue circles) and microscopy (LM as black square) results of the Bray–Curtis dissimilarity at the stress of 0.01 level. IG, inner group; MG, middle group; OG, outer group.

The results of the MDS analysis of distributions of the OTUs based on the NGS and morphology in the group samples are shown in Fig. 4. The dinoflagellate cysts identified using NGS were clearly divided into two groups (similarity = 76%) associated with years (2013 and 2014) and merged into a single group (70%). Based on microscopical analysis, they could be divided into two groups (IG-, MG-, OG-2013 and MG-, OG-2014; IG-2014) (76%) and merged into a single group (65%).

### Presence of *Cochlodinium polykrikoides* cyst

**Figure 5:**
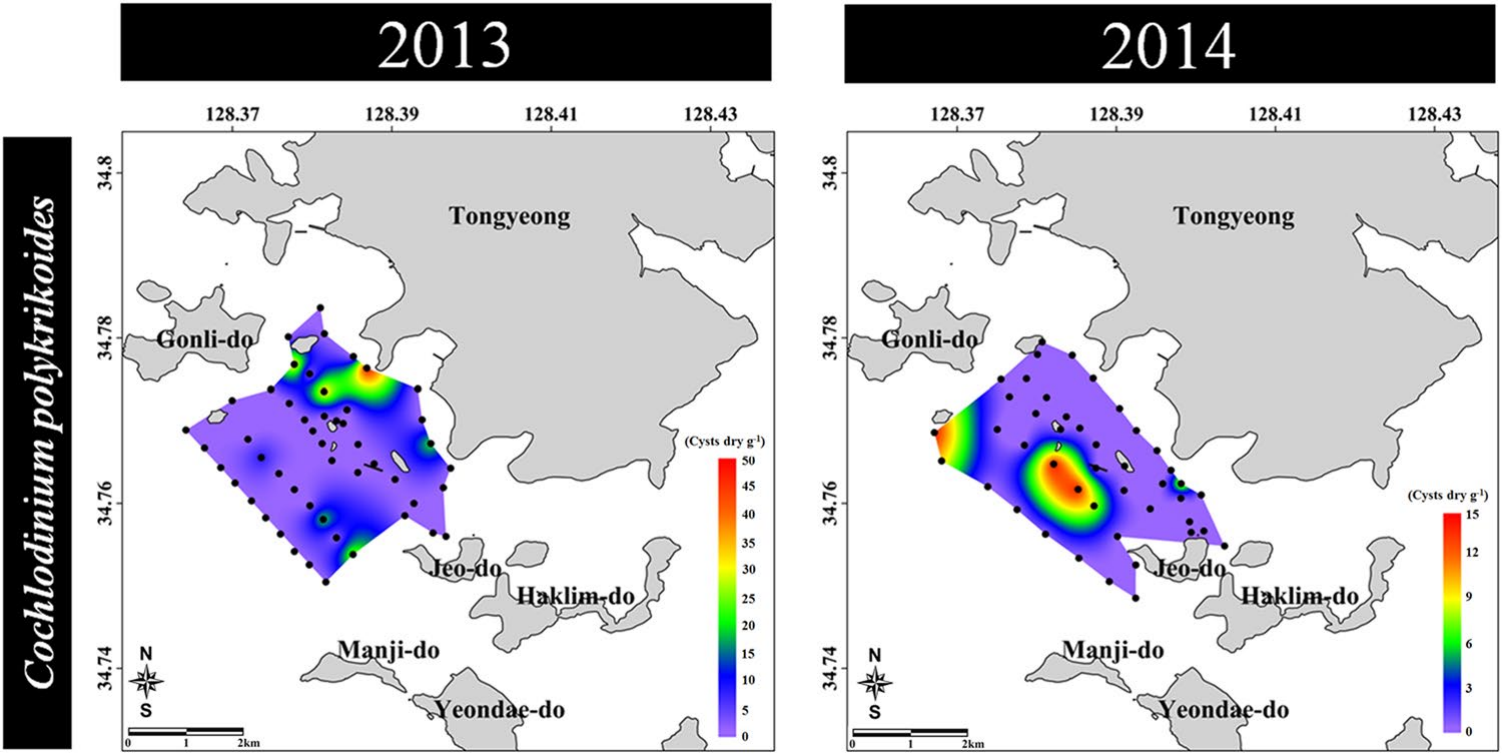
Cyst distribution of *Cochlodinium polykrikoides* (revealed by morphological analysis) in surface sediments of Tongyeong coast (South Sea) of South Korea during December 2013 (50 sampling sites) and September 2014 (44 sampling site). All maps were generated with Surfer v12.2.705 (http://www.goldensoftware.com/products/surfer).

*C. polykrikoides* bloomed in late July, peaked in early August, sustained in mid-August, and gradually decreased in late August of 2013 (Supplementary Fig. S3). In particular, the greatest density of this species was detected at density of 5,780 cells mL^−1^ on August 6, 2013. The blooming patterns of *C. polykrikoides* in 2014 were similar to that in 2013, but their strength was weaker than in 2013. Cysts of *C. polykrikoides* were not detected by NGS analysis (Supplementary Tables S1 and S2) and all PCR results were negative except for sample from the cultured *C. polykrikoides* (Supplementary Fig. S4). However, in microscopic analysis, the cysts of *C. polykrikoides* were observed at seven sampling sites (7–48 cysts g^−1^ dry weight in 2013; average cyst density of 4 cysts g^−1^ dry weight across total sampling sites), and five sampling sites had an abundance of 8–14 cyst g^−1^ dry weight in 2014 (average = 2 cysts g^−1^ dry weight) (Fig. 5).

## Discussion

Recently environmental DNA(eDNA) has begun to be employed in aquatic environmental research^12^, and it is also being used to monitor marine biodiversity^13,14^. However, we are unaware of any reports that have attempted to use eDNA to monitor dinoflagellate cysts in surface sediments. Approximately 200 dinoflagellate species produce resting cysts during their life cycle^15^ and these are deposited in the sediments. The cyst population can serve as inocula for the recurrence of blooms of harmful plankton species^2^. The dinoflagellate cysts in samples from surface sediments of the Tongyeong coast revealed a high abundance and diverse species composition (Supplementary Table S3). It is well known that dinoflagellate cysts occur at higher concentrations in muddy sediments^10^. Muddy particles are present in the surface sediments of Tongyeong coast^10,16^. Geological characteristics, such as currents along coastlines, and also determine the abundance of cysts^17^. As showing the characteristics of currents along Tongyeong coasts^18^ the cysts in Tongyeong coastal area may be introduced by currents. Moreover, Tongyeong area is one of the richest ecosystems and highest primary productivities in the Korean coast^19,20^. Thus, the abundance of dinoflagellate cysts in Tongyeong coastline is favoured by marine characteristics, such as the effects of sediment type, currents, and nutrient richness.

Small and orthoperidinoid dinoflagellate species were the most common. Cysts of *S. trochoidea* (the most predominant cyst) and *Pentapharsodinium dalei* in morphological results occurred at average proportions of 25.4 and 5.9%, respectively (Supplementary Table S4). These cysts are the most frequently encountered of the potentially harmful species. In particular, *S. trochoidea* is a cosmopolitan dinoflagellate species in coastal waters and distributed worldwide. Late summer–autumn peaks of calcareous cyst production were recorded in the Gulf of Naples^21^, and early autumn peaks of *S. trochoidea* were recorded in Onagawa Bay in Japan^4^. Cysts belonging to potentially toxic species of the genus *Alexandrium*, which cause paralytic shellfish poisoning, were recorded in 11.9% of the sediment samples. These taxa are commonly found in coastal zones across the world^22^. In the study area during 2013–2014, vegetative *S. trochoidea* and *A. catenella*/*tamarense* were frequently observed by two samplings per month at a high average abundance of 232 and 477 cells mL^−1^, respectively (unpublished data). In particular, they occurred frequently in spring and summer. In the NGS analysis, the assemblage composition is dominated by the *Pentapharsodinium*-*Scrppsiella* group, *Gonyaulax spinifera*, *Woloszynskia* group, *Alexandrium* group, and *Gymnodinium aureolum*. These dominant species detected by NGS were consistent with the morphological results. However, *Pentapharsodinium* spp. and some *Scrppsiella* spp. showed very high genetic similarity. Many researchers^23–26^ reported that *Pentapharsodinium* is a sister group or primitive lineage of *Scrippsiella* species based on molecular phylogeny of Calciodinellaceae. In this study, NGS analysis detected 35 taxa compared to only 27 cysts identified using microscopic observation. Thus, NGS analysis can allow researchers to detect higher number of species than microscopic analysis. This is because cysts have high morphological similarity and therefore are difficult to discriminate.

*C. polykrikoides* causes red tides in Tongyeong coast almost every year^7^. In particular, they developed massive blooms with density greater than 5,000 cells mL^−1^ in summer of 2013 (Supplementary Fig. S3). In our previous study, one of the several cysts isolated from surface sediments in the same study area germinated into *C. polykrikoides* cells, which was confirmed via 28S rDNA sequences^9^. In the present study, the cysts of *C. polykrikoides* were detected at ranges between 7 and 48 cysts g^−1^ dry weight based on morphological analysis. However, the cysts could not be detected in the NGS and PCR analyses. We could not perform replicate NGS analyses because the amount of gDNA of dinoflagellate cysts in the surface sediment was small. This is a weak point in the present study. This is very important to obtain accurate result and to evaluate the reproducibility of NGS for the identification of species, in particular rare species such as *C. polykrikoides*. Park *et al*.^27^ detected *C. polykrikoides* cysts by quantitative PCR using surface sediments of the same study area, but they found only five cysts in 93 samples. This result is similar to our microscopic findings. Thus, the non-detection of *C. polykrikoides* in our NGS and conventional PCR analyses could likely be due to the presence of very low amounts of *C. polykrikoides* cysts in the surface sediments. Tang and Gobler^8^ found resting cysts of *C. polykrikoides* in culture. If *C. polykrikoides* can cause massive blooms in summer, high abundance of cysts can be expected after termination of the blooms. However, relatively low densities of *C. polykrikoides* cysts were detected as compared with the massive blooms of the vegetative cells. In our preliminary study, NGS analysis of the sediments below the surface layer revealed a high proportion of *C. polykrikoides* cysts (>70%; Supplementary Fig. S5). However, in the preliminary study extraction of genomic DNA was carried out immediately after sampling, without long-term storage for six months. The high proportions of *C. polykrikoides* might be attributed to the presence of submerged dead *C. polykrikoides* and DNA debris by decomposition of *C. polykrikoides* vegetative cells, although cysts might have constituted a small portion (Fig. 5). Hattenrath-Lehmann *et al*.^28^ reported that low cyst densities after high-density blooms may be affected by several physical, chemical, and biological factors, which suggest that a small portion of the vegetative population is involved in sexual reproduction^22^. Moreover, the other factors affecting density of cysts include grazing of cysts in sediments^29^, bacterial degradation of cysts^8^, and/or the dispersal of cysts/vegetative cells out of the bloom region via currents^30^, followed by deposition of these cysts elsewhere^31^.

Evidently, the patterns of abundance of *C. polykrikoides* cysts in the present study cannot represent the prior blooming and might not predict future blooms. Thus, how blooms develop in spite of lower density of *C. polykrikoides* cysts remains unclear. *Alexandrium fundyense*, a cyst-forming dinoflagellate, can produce massive blooms from low cyst densities^22,32^. Hattenrath-Lehmann *et al*.^28^ suggested that the modest densities of cysts are important as ‘seed banks for initiating blooms’. However, the present results indicate some distinct patterns of *C. polykrikoides* cysts, as reported by Hattenrath-Lehmann *et al*.^24^. According to the hypothesis of Hattenrath-Lehmann *et al*.^28^, vegetative cells of *C. polykrikoides* were present during 2015, as evident from observation of the sediments in Tongyeong area in 2014. However, vegetative cells of *C. polykrikoides* were not detected during the summers of 2015 and 2017 (Supplementary Fig. S3). Matsuoka *et al*.^30^ and Jeong *et al*.^33^ emphasised that *C. polykrikoides* blooms developed after accumulation of cysts transported from other places. In particular, Lee *et al*.^7^ and Park *et al*.^34^ mentioned that the mechanism of bloom initiation of *C. polykrikoides* might be related to the transport of cells into the study area via lateral mixing of water of this area with that of offshore currents containing a high density of *C. polykrikoides* cells. Thus, the presence of *C. polykrikoides* might be determined by currents transporting them to a marine environment suitable for their growth.

## Methods

### Study area and sample collection

**Figure 6:**
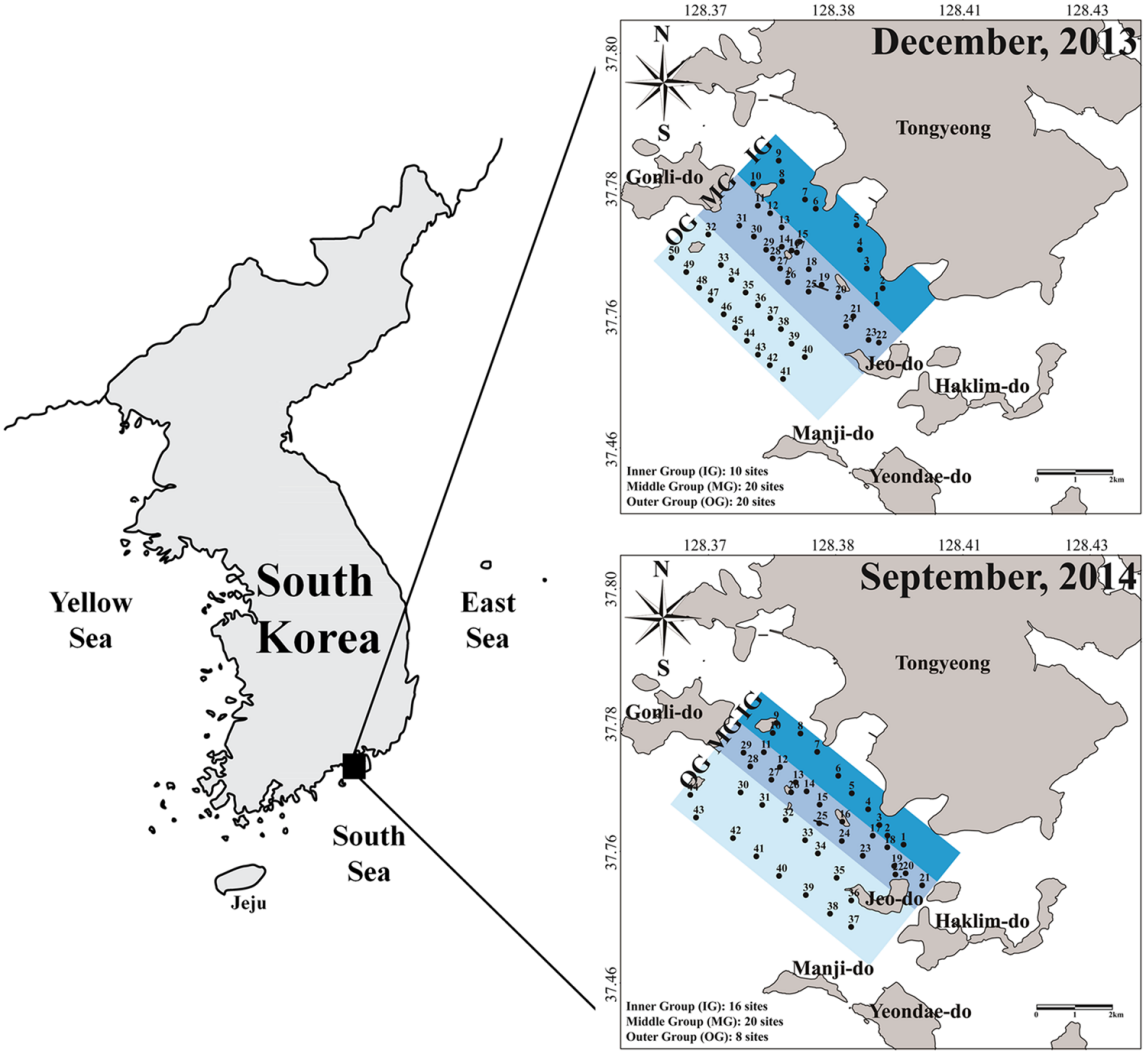
Map showing the sampling sites of surface sediments in Tongyeong coast (South Sea) of South Korea during December 2013 and September 2014. IG, inner group; MG, middle group; OG, outer group. All maps were generated with Surfer v12.2.705 (http://www.goldensoftware.com/products/surfer).

The study area is located in Tongyeong coastal waters in the southern coast of South Korea (34°45′N and 128°23′E). This costal water is a eutrophic system that is subjected to strong mixing between the surface and bottom layers, in addition to supplementation of nutrients by food supply from fisheries. Currents in the studied area are not strong, because water depths are generally shallow (<20 m), and many islands are present. In order to analyse the distribution and composition of the dinoflagellate cysts morphologically and using NGS technology, we sampled the surface sediments from 50 sites during the winter, in December 2013 (i.e. after termination of *C. polykrikoides* bloom) and 44 sites during summer in September 2014 (i.e. during the blooming period of *C. polykrikoides*) using a gravity core sampler (diameter: 5 cm). The top 2 cm of the core samples were sliced and preserved immediately in the dark at 4 °C (Fig. 6).

### Morphological analysis

The core samples for morphological analysis were collected as described by Li *et al*.^9^. One gram of sample was put into a beaker with pure seawater and processed with an ultrasonic probe for 30 s, followed by sieving with 125- and 10-μm stainless steel mesh. The residues on the 10-μm mesh stainless steel mesh were distributed equally in 10-mL aliquots with pure seawater. Dinoflagellate cysts were observed on a 1-mL sub-sample of the 10-mL aliquots in Sedgwick-Rafter counting chamber under a light microscope (Zeiss, Axio Imager A2, Hallbergmoos, Germany), and then photographed using an AxioCam MRm digital camera (Zeiss). Ten grams of sample was weighed in wet condition and dried in an oven to 110 °C for 1 day to measure the water content, and cyst concentrations in this study were estimated as the sum of living and empty cysts, which were presented as the number of cysts per gram of dry sediment.

### Genomic DNA extraction and amplification of 18S rDNA

The core samples were stored in dark and cool conditions (at 4 °C) for at least six months for decomposition of organic matter and organisms. Ten grams (wet weight) of the stored samples from each site were dried at 60 °C for 1 d in a drying oven. Thereafter, the dried sub-samples (2 g dry weight) from approximately 10–20 sites were merged and divided into the inner group (IG), middle group (MG), and outer group (OG) from the inner to outer sea, for the 44–50 sampling sites in the survey area (Fig. 6). To remove large-sized particles, the samples were subjected to a prefiltering step using a stainless steel mesh 80-μm pores. The samples after the removal of large-sized particles were harvested onto a collection filter with 10-μm pores (TCTP04700, Millipore, Billerica, MA, USA) and to remove DNA debris from the samples, filtered sediments were washed with hot sterilised seawater at approximately 70 °C and approximately 60-kPa pressure. Then, the harvested samples after removal of large particles and DNA debris were frozen at −80 °C before DNA extraction.

To analyse dinoflagellate cysts, the 18S rDNA V4-V5 gene was targeted for the NGS analysis. The genomic DNA from cysts collected was extracted using beads in a Power Soil DNA Isolation kit (MoBio, Laboratories, Solana Beach, CA, USA) and diluted to a final concentration of 20 ng μL^−1^. Amplification of the 18S rDNA gene was performed using 25-μL reaction mixtures containing 200 μmol L^−1^ each dNTP, 1.5 mmol L^−1^ MgCl_2_, 0.3 μmol L^−1^ each primer, 2.5 U Taq DNA polymerase (TaKaRa, EX Taq, Kyoto, Japan), and 1 μL DNA template. The V4–V5 region of the 18S rDNA gene was targeted using the universal primers TAReuk454FWD1 (5′-CCA GCA SCY GCG GTA ATT GG-3′) and TAReukREV3 (5′-ACT TTC GTT CTT GAT YRA-3′)^35^. Three PCR reactions were performed in distinct tubes, and then mixed to obtain more accurate NGS results. The target region (V4–V5) was chosen because of its high coverage of almost all phyla in conventional and metagenomic studies^36^. Each primer was tagged using Illumina pre-adapter and locus-specific primers following the manufacturer’s instructions (Mi-Seq, Illumina, San Diego CA, USA). The reaction profile consisted of an initial denaturation step at 95 °C for 3 min, followed by 35 cycles of denaturation at 95 °C for 10 s, annealing at 52 °C for 45 s, and extension at 72 °C for 1 min, and a final extension step of 72 °C for 5 min. Under these conditions, 800–1,000 ng PCR product was obtained using a BioRad T100 thermal cycler (BioRad, Hercules, CA, USA). Products from triplicate reactions were pooled and electrophoresed in 1.0% agarose gels, stained with ethidium bromide, and visualized under ultraviolet transillumination. Before NGS analysis, the amplified PCR products were individually purified using an UltraClean PCR Clean-up Kit (MoBio), and their DNA concentration was measured in a Bio-analyzer 2100 (Agilent Technologies, Palo Alto, CA, USA).

### Bioinformatics and data analysis

Similar amounts of PCR product from each sample were analysed using a Mi-Seq platform. The data were pre-processed using MiSeq Control Software (MCS) v2.4.1. Raw sequences were first analysed using FastQC^37^ to check basic statistics, such as GC%, per base quality score distribution, and poor-quality sequences were flagged. Also, ambiguous and chimeric reads also were removed and noised sequences (denoising), which involved OTUs with read 1, 2, and 3, were removed at cut-off of 97% (Supplementary Table S5). The processed pair-end reads were then merged using the fast length adjustment of short reads (FLASH) software tool^38^. After each sequencing procedure, a quality check was performed to remove short sequence reads (<150 bp), low-quality sequences (score <33), singletons, non-target sequences. Using Basic Local Alignment Search Tool (BLAST)^39^, all of the sequence reads were compared with those at the National Center for Biotechnology Information (NCBI) database. Sequence reads with an E-value <0.01 were considered for further analysis. A pairwise global alignment was performed on selected candidate hits to find the most similar sequences. The taxonomy of the sequence with the highest similarity was assigned to the sequence read (species or genus levels with >97% or > 94% similarity, respectively). To analyse operational taxonomic units (OTUs), the CD-HIT-OTU software^40^ was used for clustering, and metagenomic functional information to calculate alpha-diversity, including Shannon-Weaver diversity, Chao richness, and Simpson evenness based on the OTU table was generated using the closed-reference protocol in Mothur^41^ and QIIME^42^. All the six datasets were deposited in the Sequence Read Archive database at the NCBI under accessions SRS2412382 (PRJNA397358).

To test the significance of similarity between the six sequenced samples, the multidimensional scaling (MDS) analysis was performed using PRIMER 6^43^ based on Bray-Curtis distance matrices obtained from the read count of OTUs.

## Electronic supplementary material

Supplementary information
