# Supplementary material for: Mapping distribution of cysts of recent dinoflagellate and Cochlodinium polykrikoides using next-generation sequencing and morphological approaches in South Sea, Korea
Source: Sci Rep. 2018 May 3;8:7011. doi: 10.1038/s41598-018-25345-4 (PMC5934394; doi:10.1038/s41598-018-25345-4)
Supplement: Supplementary file 1 — Supplementary information [file 41598_2018_25345_MOESM1_ESM.docx]

**Supplementary Information**

Mapping distribution of cysts of recent dinoflagellate and *Cochlodinium polykrikoides* using next-generation sequencing and morphological approaches in South Sea, Korea

Seung Won Jung^1,*^, Donhyug Kang^2^, Hyun-Jung Kim^1^, Hyeon Ho Shin^1^, Joon Sang Park^3^, So Yun Park^4^, Taek-Kyun Lee^4,**^

^1^Library of Marine Samples, Korea Institute of Ocean Science & Technology, Geoje, 53201, Republic of Korea

^2^Maritime Security Research Center, Korea Institute of Ocean Science & Technology, Busan 49111, Republic of Korea

^3^Marine Ecosystem and Biological Research Center, Korea Institute of Ocean Science & Technology, Busan 49111, Republic of Korea

^4^South Sea Environment Research Center, Korea Institute of Ocean Science & Technology, Geoje 53201, Republic of Korea

^*^Corresponding author: S.-W. Jung, diatoms@kiost.ac.kr, Fax: +82-55-639-8429

^**^ Co-corresponding author: T.K. Lee, tklee@kiost.ac.kr, Fax: +82-55-639-8429


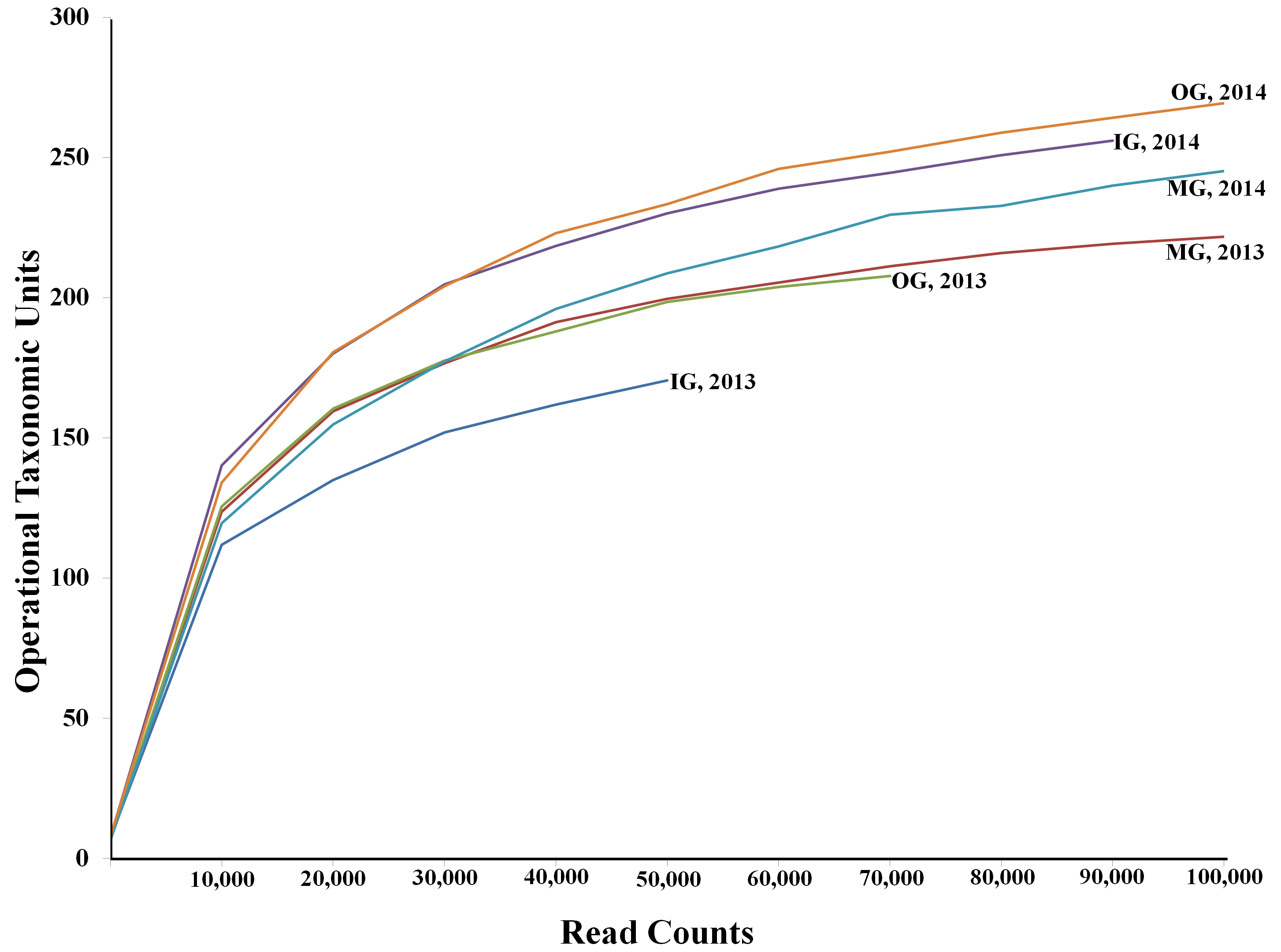


**Supplementary Figure S1.** Rarefaction curves of the next-generation sequencing data in surface sediments in of Tongyeong coast (South Sea) of South Korea during December 2013 (50 sampling sites) and September 2014 (44 sampling site). IG, inner group; MG, middle group; OG, outer group.


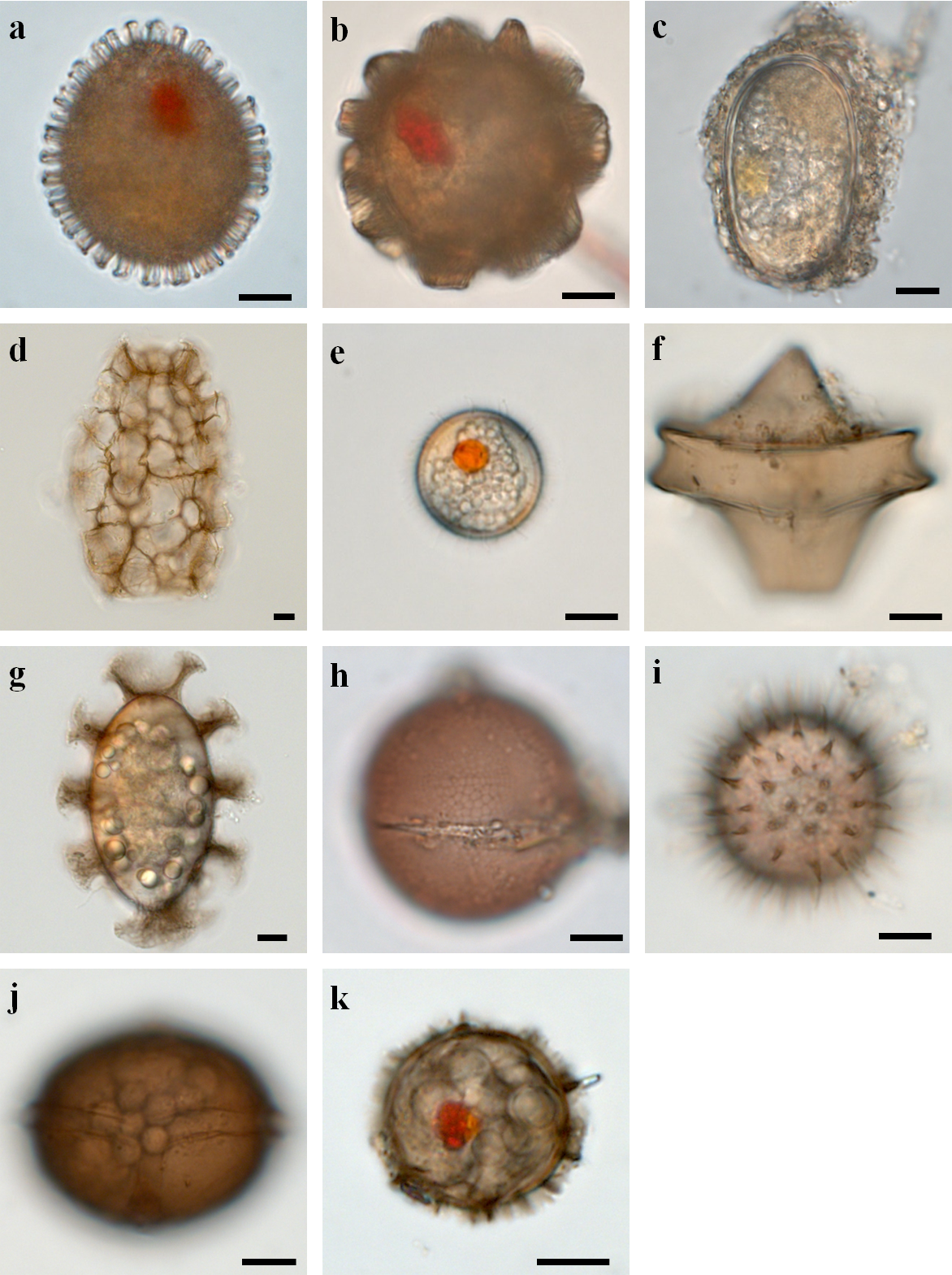


**Supplementary Figure S2.** Microscopic photographs of common dinoflagellate cysts in surface sediments of Tongyeong coast (South Sea) of South Korea during December 2013 and September 2014. a, *Scrippsiella trochoidea*; b, *Ensiculifera carinata*; c, *Alexandrium catenella/tamarense* type; d, *Polykrikoides schwartzii*; e, *Pentaphasodinium dalei*; f, *Selenopemphix nephroides*; g, *Polykrikoides kofoidii*; h, *Gymnodinium catenatum*; i, *Oblea acanthocysta*; j, *Protoperidinium meunieri*; k, *Cochlodinium polykrikoides* . Scale bars: 10 μm.

**Supplementary Figure S3.** Occurrence of vegetative *Cochlodinium polykrikoides* red tide (revealed by microscopic analysis) in Tongyeong coastal waters from 2013–2017.


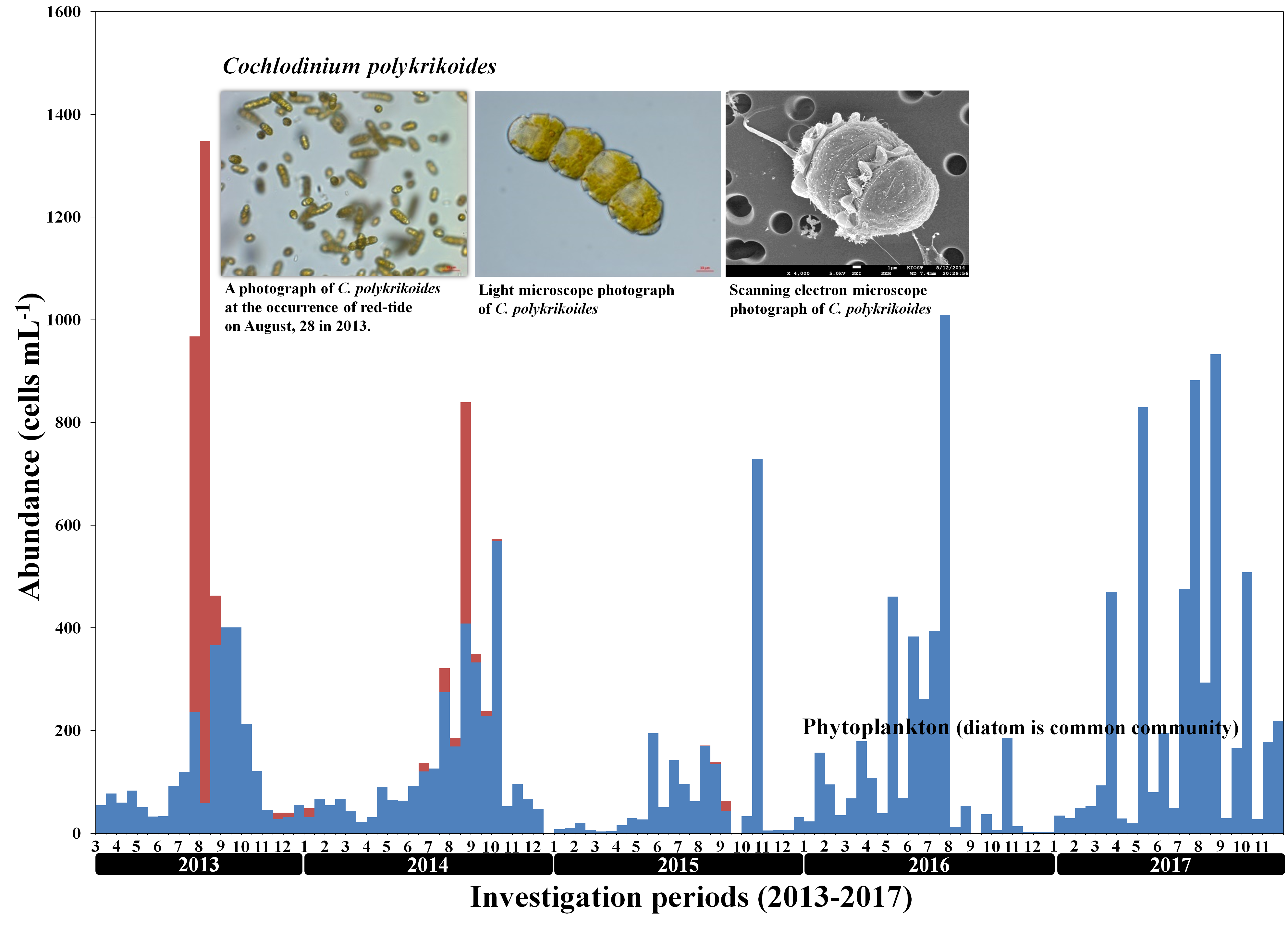


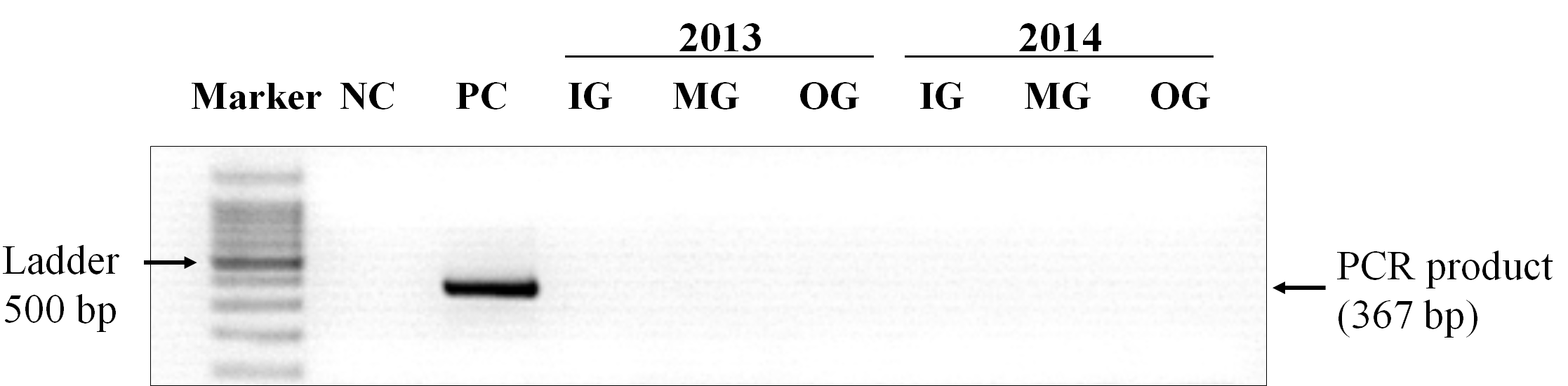


**Supplementary Figure S4.** Electrophoresis of the nested PCR product for the detection of large subunit ribosomal DNA (LSU rDNA) gene of *C. polykrikoides* in surface sediments of Tongyeong coast (South Sea) of South Korea. DNA marker; 100 bp, NC; negative control or no template control, PC; positive control, six field samples of inner group (IG), middle group (MG), and outer group (OG) of 2013 and 2014. Primers of the LSU rDNA used in this study have been previously described by Park *et al.*^1^.

**Analyzed method:** The core samples were stored in dark and cool conditions (at 4^°^C) for at least six months for decomposition of organic matter and organisms. Ten grams (wet weight) of the stored samples from each site were dried at 60°C for 1 d in a drying oven. Thereafter, the perfectly dried sub-sample (2 g dry weight) from approximately 10–20 sites were merged and divided into the inner group (IG), middle group (MG), and outer group (OG) from the inner to outer sea of total 44-50 sampling sites in the survey area (Fig. 1). To remove large-sized particles, the samples were subjected to a prefiltering step using a stainless steel mesh 80-μm pores. The samples after the removal of large-sized particles were harvested onto a collection filter with 10-μm pores (TCTP04700, Millipore, Billerica, MA, USA) and to remove DNA debris from the samples, filtered sediments were washed with hot sterilised seawater at approximately 70°C and approximately 60-kPa pressure. Thus, the harvested samples after removal of large particles and DNA debris were frozen at -80°C before DNA extraction.

Nested PCR was performed for detection of large subunit ribosomal DNA (LSU rRNA) gene of *C. polykrikoides*. The genomic DNA from cysts collected was extracted using beads in a Power Soil DNA Isolation kit (MoBio, Laboratories, Solana Beach, CA, USA) and diluted to a final concentration of 20 ng μL^−1^. Amplification of the 28S rDNA partial gene was performed using 25-μL reaction mixtures containing 200 μmol L^−1^ each dNTP, 1.5 mmol L^−1^ MgCl_2_, 0.3 μmol L^−1^ each primer, 2.5 U Taq DNA polymerase (TaKaRa, EX Taq, Kyoto, Japan), and 1 μL DNA template. The partial region of the 28S rDNA gene was targeted using the specific primers of *C. polykrikoides* CPLSUF (5’-GCCGAGGATACCTGCAAAG-3’) and CPLSUR (5’-TGTCAGGACCCACGATCA-3’) Primers of the LSU rRNA used in this study have been previously described (Park *et al.* 2016). For the first PCR, 5 min at 94°C and then 35 cycles of 30 sec at 94°C, 30 sec at 60°C, and 1 min at 72°C with a terminal step of 5 min at 72°C and then the mixture was held at 4°C. For the second PCR, 2 μl of the first-round PCR product was used. As positive PCR controls, genomic DNA from the cultured *C. polykrikoides* was used as templates. Finally, the PCR products were visualized by ethidium bromide staining after electrophoresis in 2 % agarose.


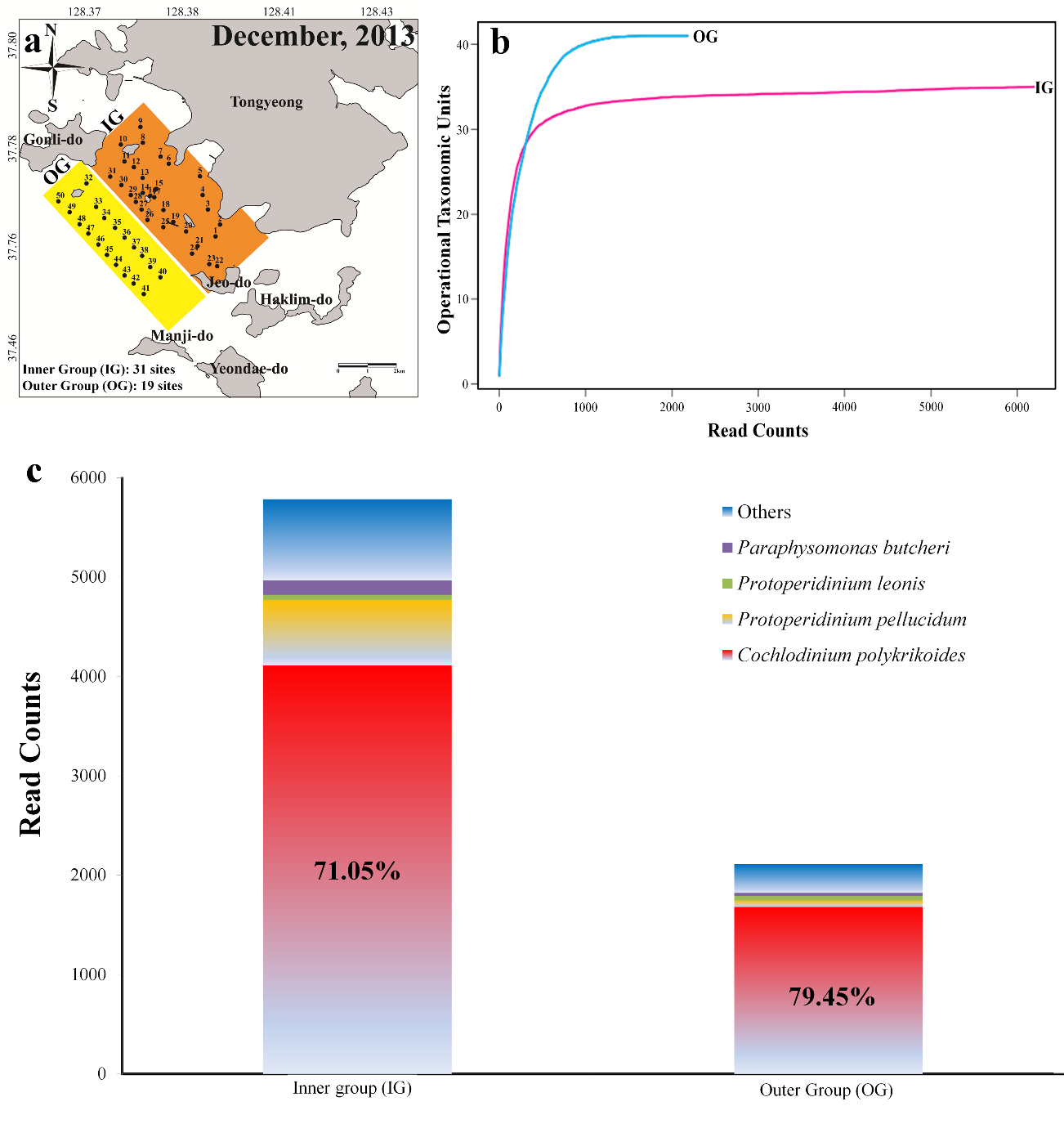


**Supplementary Figure S5.** Presence of *Cochlodinium polykrikoides* (revealed by next-generation sequencing analysis) in surface sediments of Tongyeong coast (South Sea) of South Korea, 2013. Immediately after sampling, the samples were analysed by next-generation sequencing after extracting the genomic DNA. (a) Map showing the sampling sites of surface sediments. (b) Rarefaction curves of the next-generation sequencing data, and (c) results of next-generation sequencing analysis. The map is generated with Surfer v12.2.705 (http://www.goldensoftware.com/products/surfer).

**Supplementary Table S1.** Read counts and similarity of common dinoflagellate cysts (at mean proportion of over 1%) in surface sediments of Tongyeong coast (South Sea) of South Korea during 2013 and 2014. IG, inner group; MG, middle group; OG, outer group.

| Presented cysts | 2013 | | | | 2014 | | | | Similarity (%) | Max score | Accession number | References |
| --- | --- | --- | --- | --- | --- | --- | --- | --- | --- | --- | --- | --- |
|  | IG | MG | OG | mean | IG | MG | OG | mean |  |  |  |  |
| *Pentapharsodinium-Scrppsiella* complex | 1907 | 3094 | 1916 | 2306 | 1771 | 1699 | 2369 | 1946 |  |  |  |  |
| *Pentapharsodinium tyrrhenicum* |  |  |  |  |  |  |  |  | 100% | 774 | AF022201 | 2 |
| *Pentapharsodinium dalei* |  |  |  |  |  |  |  |  | 99% | 769 | JX262492 | 3 |
| *Scrippsiella trochoidea* |  |  |  |  |  |  |  |  | 99% | 763 | KF733540 | 4 |
| *Scrippsiella sweeneyae* |  |  |  |  |  |  |  |  | 99% | 763 | HQ845331 | 5 |
| *Scrippsiella erinaceus* |  |  |  |  |  |  |  |  | 99% | 756 | KJ189477 | 6 |
| *Gonyaulax spinifera* | 81 | 135 | 192 | 136 | 176 | 549 | 442 | 389 | 96% | 680 | AF022155 | 7 |
| *Alexandrium* complex I | 150 | 432 | 84 | 222 | 67 | 299 | 280 | 215 |  |  |  |  |
| *Alexandrium pacificum* |  |  |  |  |  |  |  |  | 100% | 760 | KF908800 | 8 |
| *Alexandrium catenella* |  |  |  |  |  |  |  |  | 100% | 760 | KM091276 | 9 |
| *Alexandrium tamarense* |  |  |  |  |  |  |  |  | 100% | 760 | KF733551 | 10 |
| *Alexandrium fundyense* |  |  |  |  |  |  |  |  | 99% | 732 | KF908796 | 8 |
| *Woloszynskia* complex | 150 | 328 | 200 | 226 | 76 | 242 | 72 | 130 |  |  |  |  |
| *Woloszynskia cincta* |  |  |  |  |  |  |  |  | 100% | 760 | JN934667 | 10 |
| *Woloszynskia halophile* |  |  |  |  |  |  |  |  | 100% | 760 | EF058252 | 11 |
| *Alexandrium* complex II | 47 | 272 | 84 | 134 | 134 | 53 | 159 | 115 |  |  |  |  |
| *Alexandrium minutum* |  |  |  |  |  |  |  |  | 99% | 743 | JF521635 | 12 |
| *Alexandrium insuetum* |  |  |  |  |  |  |  |  | 99% | 732 | JF521630 | 12 |
| *Alexandrium tamutum* |  |  |  |  |  |  |  |  | 99% | 732 | AJ535379 | 13 |
| *Alexandrium lusitanicum* |  |  |  |  |  |  |  |  | 99% | 726 | JF906999 | 14 |
| *Gyrodinium impudicum* | 94 | 108 | 43 | 82 | 31 | 30 | 30 | 30 | 99% | 737 | AF022197 | 7 |
| *Scrippsiella hangoei* | 49 | 71 | 116 | 79 | 25 | 15 | 15 | 18 | 98% | 693 | EF417316 | 15 |

**Supplementary Table S2.** Read counts and similarity of rare dinoflagellate cysts (at mean proportion of less 1%) in surface sediments of Tongyeong coast (South Sea) of South Korea during 2013 and 2014.

| Presented cysts | 2013 | | | | 2014 | | | | Similarity (%) | Max score | Accession number | References |  |
| --- | --- | --- | --- | --- | --- | --- | --- | --- | --- | --- | --- | --- | --- |
|  | IG | MG | OG | mean | IG | MG | OG | mean |  |  |  |  |  |
| *Alexandrium leei* | 0 | 0 | 17 | 6 | 0 | 0 | 0 | 0 | 98% | 693 | AY641565 | 16 |  |
| *Alexandrium* complex III | 0 | 67 | 0 | 22 | 0 | 0 | 16 | 5 |  |  |  |  |  |
| *Alexandrium hiranoi* |  |  |  |  |  |  |  |  | 99% | 743 | LC056070 | 17 |  |
| *Alexandrium pseudogoniaulax* |  |  |  |  |  |  |  |  | 99% | 743 | JF521638 | 18 |  |
| *Alexandrium taylori* |  |  |  |  |  |  |  |  | 99% | 737 | AJ535390 | 13 |  |
| *Archaeperidinium* complex | 7 | 0 | 0 | 2 | 1 | 0 | 1 | 1 |  |  |  |  |  |
| *Archaeperidinium minutum* |  |  |  |  |  |  |  |  | 95% | 623 | AB564309 | 18 |  |
| *Archaeperidinium saanichi* |  |  |  |  |  |  |  |  | 95% | 617 | AB702987 | 19 |  |
| *Duboscquella* sp. | 0 | 0 | 0 | 0 | 6 | 3 | 5 | 5 | 97% | 665 | JF791082 | 2 |  |
| *Fragilidium mexicanum* | 14 | 22 | 36 | 24 | 0 | 3 | 7 | 3 | 99% | 734 | FJ405355 | 20 |  |
| *Gonyaulax cochlea* | 0 | 1 | 0 | 0 | 28 | 0 | 60 | 29 | 88% | 484 | DQ388465 | 21 |  |
| *Gonyaulax verior* | 0 | 28 | 5 | 11 | 0 | 8 | 0 | 3 | 99% | 736 | AY443013 | 22 |  |
| *Gymnodinium aureolum* | 96 | 196 | 97 | 130 | 76 | 92 | 77 | 82 | 98% | 699 | KJ481834 | 23 |  |
| Gymnodinium sp. | 0 | 0 | 0 | 0 | 15 | 6 | 10 | 10 | 94% | 604 | AB860180 | 24 |  |
| *Gymnodinium impudicum* | 28 | 52 | 21 | 34 | 5 | 15 | 7 | 9 | 99% | 737 | DQ785884 | 25 |  |
| *Scrippsiella* sp. | 10 | 0 | 0 | 3 | 5 | 4 | 5 | 5 | 97% | 675 | LC054940 | 17 |  |
| *Polykrikos geminatum* | 14 | 46 | 4 | 21 | 21 | 30 | 21 | 24 | 93% | 588 | JX967270 | 26 |  |
| *Protoperidinium* complex | 10 | 10 | 25 | 15 | 11 | 15 | 19 | 15 |  |  |  |  |  |
| *Protoperidinium tricingulatum* |  |  |  |  |  |  |  |  | 99% | 737 | AB716918 | 27 |  |
| *Protoperidinium fukuyoi* |  |  |  |  |  |  |  |  | 99% | 715 | AB780842 | 28 |  |
| *Protoperidinium parthenopes* |  |  |  |  |  |  |  |  | 98% | 710 | AB716915 | 29 |  |
| *Protoperidinium americanum* |  |  |  |  |  |  |  |  | 98% | 710 | AB716911 | 29 |  |
| *Oblea acanthocysta* | | 0 | 99 | 73 | 57 | 0 | 5 | 0 | 2 | 97% | 678 | LC005409 | 30 |
| *Polykrikos-Pheopolykrikos* complex | | 5 | 30 | 18 | 18 | 8 | 13 | 14 | 12 |  |  |  |  |
| *Polykrikos geminatum* | |  |  |  |  |  |  |  |  | 99% | 715 | JX967270 | 26 |
| *Polykrikos tanit* | |  |  |  |  |  |  |  |  | 98% | 710 | KF806599 | 31 |
| *Pheopolykrikos hartmanii* | |  |  |  |  |  |  |  |  | 98% | 704 | AY421789 | 32 |
| *Polykrikos schwartzii* | | 36 | 8 | 2 | 15 | 18 | 32 | 37 | 29 | 99% | 737 | AB466287 | 33 |
| *Prorocentrum* complex | | 4 | 0 | 8 | 4 | 2 | 0 | 1 | 1 |  |  |  |  |
| *Prorocentrum koreanum* | |  |  |  |  |  |  |  |  | 98% | 684 | KP711353 | 34 |
| *Prorocentrum micans* | |  |  |  |  |  |  |  |  | 98% | 684 | KP711341 | 34 |
| *Protoceratium reticulatum* | | 0 | 2 | 0 | 1 | 13 | 9 | 3 | 8 | 99% | 736 | AB727656 | 35 |
| *Protoperidinium claudicans* | | 14 | 37 | 5 | 19 | 0 | 10 | 2 | 4 | 100% | 749 | AB255833 | 36 |
| *Protoperidinium minutum* | | 7 | 8 | 11 | 9 | 17 | 10 | 16 | 14 | 99% | 737 | GQ227501 | 37 |
| *Protoperidinium monovelum* | | 35 | 99 | 51 | 62 | 1 | 1 | 14 | 5 | 95% | 634 | AB716913 | 29 |
| *Spatulodinium pseudonoctiluca* | | 0 | 0 | 0 | 0 | 7 | 4 | 2 | 4 | 100% | 739 | GU355684 | 38 |
| *Suessiaceae* sp. | | 15 | 1 | 3 | 6 | 0 | 11 | 19 | 10 | 91% | 545 | LC068841 | 39 |
| Unidentified dinoflagellate cyst complex Ⅰ | | 7 | 24 | 13 | 15 | 3 | 11 | 3 | 6 |  |  |  |  |
| *Parvilucifera prorocentri* | |  |  |  |  |  |  |  |  | 89% | 501 | FJ424512 | 40 |
| Uncultured *Gyrodinium* sp. | |  |  |  |  |  |  |  |  | 89% | 492 | JQ692033 | 41 |
| Unidentified dinoflagellate cyst complex Ⅱ | | 0 | 20 | 4 | 8 | 4 | 4 | 12 | 7 |  |  |  |  |
| *Blastodinium mangini* | |  |  |  |  |  |  |  |  | 89% | 492 | JX473656 | 42 |
| *Heterocapsa pygmaea* | |  |  |  |  |  |  |  |  | 89% | 492 | AF274266 | 43 |
| *Azadinium cuneatum* | |  |  |  |  |  |  |  |  | 88% | 486 | KJ481822 | 23 |
| Unidentified dinoflagellate cyst complex Ⅲ | | 0 | 0 | 6 | 2 | 10 | 16 | 5 | 10 |  |  |  |  |
| *Dissodinium pseudolunula* | |  |  |  |  |  |  |  |  | 84% | 375 | FJ473378 | 44 |
| *Tintinnophagus acutus* | |  |  |  |  |  |  |  |  | 83% | 364 | HM483397 | 45 |
| Uncultured marine *Peridiniaceae* sp. | |  |  |  |  |  |  |  |  | 83% | 364 | FJ431865 | 46 |
| *Peridinium umbonatum* | |  |  |  |  |  |  |  |  | 83% | 364 | GU001637 | 47 |
| Unidentified dinoflagellate cyst complex Ⅳ | | 5 | 11 | 3 | 6 | 2 | 1 | 3 | 2 | 100% | 739 | AY775284 | 48 |
| Unidentified dinoflagellate cyst complex Ⅴ | | 0 | 0 | 0 | 0 | 1 | 7 | 1 | 3 |  |  |  |  |
| *Gyrodinium heterogrammum* | |  |  |  |  |  |  |  |  | 87% | 448 | KP790159 | 49 |
| *Gymnodinium limneticum* | |  |  |  |  |  |  |  |  | 87% | 442 | KR362900 | 50 |
| *Gyrodinium* cf. *spirale* | |  |  |  |  |  |  |  |  | 87% | 442 | KP790157 | 49 |
| *Gymnodinium palustre* | |  |  |  |  |  |  |  |  | 87% | 442 | AB921299 | 51 |

**Supplementary Table S3.** Summary of 34 references of cyst abundances and predominant cysts, worldwide.

| **Cyst Abundances**  **(cells g^-1^ dry weight)** | **No. of**  **morphotype** | **Dominant species** | **Sampling site** | **Reference** |
| --- | --- | --- | --- | --- |
| 384 - 9,944 | 42 | *Gymnodinium nolleri, Spiniferites bulloideus, Lingulodinium machaerophorum* | Izmir Bay (Aegean Sea) | 52 |
| 701-54,105 | 24 | *Pentapharsodinium dalei, Operculodinium centrocarpum, Islandinium minutum* | Hudson Bay, Hudson Strait (Inland Sea) | 53 |
| 21-322* | 62 | *Scrippsiella trochoidea complex, Biecheleria cincta, Pentapharsodinium tyrrhenicum* | Fangar Bay and Alfacs Bay (Mediterranean Sea) | 54 |
| 1,775-25,683 | 18 | *Operculodinium centrocarpum, Lingulodinium machaerophorum, Spiniferites* spp., *Pentapharsodinium dalei, Dubridinium* spp. | Kattegat and Skagerrak basins (Baltic Sea) | 55 |
| 46-2,317 | 53 | *Protoperidinium* cf *tricingulatum, Scrippsiella* spp., *S. trochoidea* complex | Cabras, Santa Giusta, and Corru S'Ittiri lagoon (Mediterranean Sea) | 56 |
| 78-3,576 | 54 | *Brigantedinium* spp., *Polysphaeridium zoharyi, Spiniferites* spp., *Operculodinium* spp. | West-central Florida coast (Gulf of Mexico) | 57 |
| 11-1,218 | 28 | *Protoceratium reticulatum, Protoperidinium leonis, P. oblongum, P.* cf. *pentagonum* | Visakhapatnam harbor (India Ocean) | 58 |
| 29-331 | 24 | *Gonyaulax spinifera* complex, *Lingulodinium polyedrum, Protoperidinium compressum* | Bengal Bay (India Ocean) | 59 |
| 8-110 | 26 | *Protoperidinium* sp., *P. subinerme, P. pentagonum* | Aalong the Port Blair (India Ocean) | 60 |
| 748–7,887 | - | *Biecheleria baltica, Peridiniella catenata, Scrippsiella hangoei* | Gulf of Finland and Riga, Baltic proper  (Baltic sea) | 61 |
| 203-2,742 | 22 | *Alexandrium pseudogonyaulax, Protoperidinium claudicans* | Bizerte Lagoon (Mediterranean Sea) | 62 |
| 0-1,660 | 40 | *Bitectatodinium spongium, Brigantedinium* spp*., Selenopemphix nephroides, S. robustum, Lejeunecysta* spp. | Bengal Bay (India Ocean) | 63 |
| 2,250 | 35 | *Spiniferites* spp., *Brigantedinium* spp., *Operculodinium centrocarpum, Pentapharsodinium dalei, Selenopemphix nephroides* | Gulf of Aqaba (Red Sea) | 64 |
| 0-229 | 16 | *Protoperidinium* spp*., Scrippsiella trochoidea* complex, *Lingulodinium machaerophorum, Alexandrium* spp. | Ghar El Melh Lagoon (Mediterranean Sea) | 65 |
| 9,106-11,815 | 25 | *Brigantedinium* spp., *Protoperidinium americanum, Alexandrium* spp., *Spiniferites* spp., *Pentapharsodinium dalei* | Patricia Bay in Saanich Inlet  (North Pacific Ocean) | 66 |
| 11-2,543 | 42 | *Gymnodinium nolleri, Alexandrium affine, Lingulodinium machaerophorum, Operculodinium centrocarpum* | Nemrut and Aliağa Bay (Aegean Sea) | 67 |
| 8-346 | 26 | *Lingulodinium machaerophorum, Alexandrium minutum, Spiniferites bulloideus* | Black Sea, Çanakkale Strait (Mediterranean Sea) | 68 |
| 34-31,532 | 38 | *Lingulodinium machaerophorum, Polykrikoids kofoidii, Quinquecuspis concreta, Dubridinium caperatum, Spiniferites bulloideus* | Izmir Bay (Mediterranean Sea) | 69 |
| 0-25,860* | 28 | *Gonyaulax spinifera, Pentapharsodinium dalei, Protoceratium reticulatum* | Bering Sea | 70 |
| 1,000-6,000 | 16 | *Pentapharsodinium dalei, Spiniferites ramosus, Islandinium minutum, Brigantedinium* spp. | Nunatsiavut (Arctic Ocean) | 71 |
| 51-695 | 22 | *Alexandrium* spp., *Brigantedinium* spp., *Operculodinium centrocarpum, Spiniferites bulloideus, Spiniferites bulloideus, Scrippsiella trochoidea, Tuberculodinium vancampoae* | Offshore South Sea (South Korea) | 72 |
| 253-5,568 | 19 | *Brigantedinium* spp., *Selenopemphix quanta, S.* cf. *S. quanta, Pentapharsodinium dalei* | Beagle Channel (Atlantic Ocean) | 73 |
| 122-1,322 | 35 | *Spiniferites bentori var. truncata, Brigantedinium* sp.1, *Quinquecuspis concreta* | Sishili Bay (Yellow Sea) | 74 |
| 13.93-39.9 | 42 | *Protoperidinium* spp., *Brigantedinium* spp., *Selenopemphix* spp. | Southern Myanmar coast (Andaman Sea) | 75 |
| 0-128 | 30 | *Pheopolykrikos hartmannii, Pyrophacus steinii* | Coastal areas of Chonburi province (Thailand) | 76 |
| 43-828 | 24 | *Alexandriumminutum, Gymnodinium nolleri, Lingulodinium polyedrum, Pentapharsodiniumtyrrhenicum, Scrippsiella trochoidea* | Syracuse Bay (Ionian Sea) | 77 |
| 1,000-8,900 | 47 | *Spiniferites* spp*., Brigantedinium* spp., *Dubridinium* spp. | South Sea coast (South Korea) | 78 |
| 66-55,620 | 34 | *Brigantedinium* spp., *Bitectatodinium spongium, Selenopemphix quanta, Polykrikoids kofoidii, Echinidinium* spp. | Mexican coast (Pacific Ocean) | 79 |
| 655-1,725 | 42 | *Brigantedinium* spp.*, Protoperidinium americanum, Polykrikos* spp., *Lingulodinium machaerophorum, Spiniferites* spp. | South Sea coast (South Korea) and Ariake Bay (Japan) | 80 |
| 480-10,679* | 42 | *Scrippsiella trochoidea, S.* sp. 2, *Gymnodiniales* type 1, *S. precaria* | Arenys de Mar harbor and Olbia Gulf (Spain) | 81 |
| 173-1,276 | 35 | *Polykrikos* spp*., Brigantedinium* spp., *Spiniferites* spp. | South Sea coast (South Korea) | 82 |
| 3-4,083 | 19 | *Cochlodinium polykrikos, Prorocentrum minimum, Dinophysis acuminate, Alexandrium catenella, Scrippsiella trochoidea* | South-western Saudi coasts (Red Sea) | 83 |
| 100-25,640 | 36 | *Brigantedinium* spp*., Echinidinium* spp*., Dubridinium* spp*., Quinquecuspis concreta, Polykrikos kofoidii, P. schwartzii* | Vancouver Island (Canada) | 84 |
| 57-929 | - | *Impagidinium aculeatum, Spiniferites* spp*., Brigantedi nium* spp. | Mediterranean Sea | 85 |

**Supplementary Table S4.** Distribution of dinoflagellate cysts using microscopic analysis in surface sediments of Tongyeong coast (South Sea) of South Korea during 2013 and 2014

| Dinoflagellate cysts | 2013 | | | | 2014 | | | |
| --- | --- | --- | --- | --- | --- | --- | --- | --- |
|  | IG | MG | OG | Mean | IG | MG | OG | Mean |
| *Scrippsiella trochoidea* | 281 | 345 | 274 | 300 | 92 | 177 | 192 | 154 |
| *Scrippsiella* spp. | 88 | 118 | 153 | 120 | 64 | 154 | 137 | 119 |
| *Ensiculifera carinata* | 82 | 111 | 72 | 88 | 23 | 124 | 139 | 96 |
| *Protoperidinium* spp. spiny type | 76 | 107 | 101 | 95 | 77 | 98 | 83 | 86 |
| *Polykrikos schwartzii* | 71 | 132 | 94 | 99 | 32 | 40 | 38 | 36 |
| *Alexandrium catenella/tamarense* type | 73 | 53 | 49 | 58 | 6 | 82 | 95 | 61 |
| *Pentapharsodinium dalei* | 102 | 90 | 101 | 97 | 0 | 14 | 14 | 9 |
| *Alexandrium minutum* type | 57 | 49 | 51 | 52 | 24 | 44 | 39 | 36 |
| *Phaeopolykrikos hartmanii* | 57 | 64 | 50 | 57 | 0 | 9 | 2 | 4 |
| *Polykrikos kofoidii* | 9 | 24 | 35 | 23 | 12 | 28 | 29 | 23 |
| *Gymnodinium catenatum* | 25 | 9 | 39 | 24 | 0 | 25 | 17 | 14 |
| *Scrippsiella tridida* | 2 | 5 | 3 | 3 | 13 | 24 | 24 | 21 |
| *Oblea acanthocysta* | 13 | 20 | 5 | 13 | 2 | 15 | 12 | 10 |
| *Scrippsiella precaria* | 7 | 5 | 1 | 4 | 0 | 24 | 24 | 16 |
| *Gymnodinium* spp. | 4 | 11 | 14 | 10 | 0 | 5 | 4 | 3 |
| *Preperidinium meunieri* | 6 | 15 | 6 | 9 | 0 | 0 | 0 | 0 |
| *Protoperidinium latissimum* | 0 | 1 | 3 | 1 | 2 | 10 | 9 | 7 |
| *Cochlodinium* sp. | 10 | 2 | 3 | 5 | 2 | 0 | 6 | 3 |
| *Scrippsiella crystallina* | 4 | 9 | 0 | 4 | 0 | 4 | 4 | 2 |
| *Cochlodinium polykrikoides* | 4 | 4 | 3 | 4 | 0 | 0 | 2 | 1 |
| *Protoperidinium leonis* | 0 | 0 | 0 | 0 | 9 | 0 | 0 | 3 |
| *Scrippsiella rotunda* | 3 | 2 | 0 | 2 | 0 | 0 | 3 | 1 |
| *Fragilidium* sp. | 2 | 0 | 0 | 1 | 2 | 3 | 0 | 2 |
| *Protoperidinium minutun* | 2 | 0 | 0 | 1 | 0 | 3 | 1 | 1 |
| *Scrippsiella operosa* | 0 | 1 | 0 | 0 | 0 | 1 | 4 | 2 |
| *Gonyaulax verior* | 2 | 2 | 3 | 2 | 0 | 1 | 0 | 0 |
| *Protoperidinium americanum* | 0 | 0 | 0 | 0 | 0 | 1 | 0 | 0 |
| unknown type | 0 | 0 | 0 | 0 | 0 | 10 | 14 | 8 |
| Total (cysts/g dry weight) | 979 | 1180 | 1057 | 1072 | 360 | 897 | 892 | 716 |

**Supplementary Table S5.** Summary of preprocessing and clustering steps of NGS analysis in surface sediments of Tongyeong coast (South Sea) of South Korea during 2013 and 2014.

| Assembled reads | Removed reads | | Remaining reads |
| --- | --- | --- | --- |
| 306,847 | Ambiguous | 22 | 56,320 |
|  | Low-Quality | 11,967 |  |
|  | Chimera | 5,450 |  |
|  | Rare cluster | 233,088 |  |

References

1. Park, T. G., Kim, J. J., Kim, W. J. & Won, K. M. Development of real-time RT-PCR for detecting viable *Cochlodinium polykrikoides* (Dinophyceae) cysts in sediment. *Harmful algae* **60**, 36-44 (2016).

2. Rocke, E., Jing, H. & Liu, H. Phylogenetic composition and distribution of picoeukaryotes in the hypoxic northwestern coast of the Gulf of Mexico. *Microbiologyopen* **2**, 130-143 (2013).

3. Orr, R. J., Murray, S. A., Stüken, A., Rhodes, L. & Jakobsen, K. S. When naked became armored: an eight-gene phylogeny reveals monophyletic origin of theca in dinoflagellates. *PloS one* **7**, e50004; 10.1371/journal.pone.0050004 (2012).

4. Hu, S. *et al.* Detecting *In Situ* copepod diet diversity using molecular technique: development of symbiotic ciliate-excluding eukaryote-inclusive PCR protocol. *PloS one* **9**, e103528; 10.1371/journal.pone.0103528 (2014).

5. Gottschling, M. *et al.* Delimitation of the Thoracosphaeraceae (Dinophyceae), including the calcareous dinoflagellates, based on large amounts of ribosomal RNA sequence data. *Protist* **163**, 15-24 (2012).

6. Kretschmann, J., Zinssmeister, C. & Gottschling, M. Taxonomic clarification of the dinophyte *Rhabdosphaera erinaceus* Kamptner, ≡ *Scrippsiella erinaceus* comb. nov. (Thoracosphaeraceae, Peridiniales). *Syst. Biodivers.* **12**, 393-404 (2014).

7. Saunders, G., Hill, D., Sexton, J. & Andersen, R. Small-subunit ribosomal RNA sequences from selected dinoflagellates: testing classical evolutionary hypotheses with molecular systematic methods. *Plant Syst. Evol.* **11**, 237–259 (1997).

8. John, U. *et al.* Formal revision of the *Alexandrium tamarense* species complex (Dinophyceae) taxonomy: the introduction of five species with emphasis on molecular-based (rDNA) classification. *Protist* **165**, 779-804 (2014).

9. Zhang, Y., Zhang, S. F., Lin, L. & Wang, D. Z. Comparative transcriptome analysis of a toxin-producing dinoflagellate *Alexandrium catenella* and its non-toxic mutant. *Mar. drugs* **12**, 5698-5718 (2014).

10. Balzano, S. *et al.* Diversity of cultured photosynthetic flagellates in the northeast Pacific and Arctic Oceans in summer. *Biogeosciences* **9**, 4553-4571 (2012).

11. Logares, R., Shalchian-Tabrizi, K., Boltovskoy, A. & Rengefors, K. Extensive dinoflagellate phylogenies indicate infrequent marine–freshwater transitions. *Mol. Phylogenet. Evol.* **45**, 887-903 (2007).

12. Orr, R. J., Stüken, A., Rundberget, T., Eikrem, W. & Jakobsen, K. S. Improved phylogenetic resolution of toxic and non-toxic *Alexandrium* strains using a concatenated rDNA approach. *Harmful Algae* **10**, 676-688 (2011).

13. John, U., Fensome, R. A. & Medlin, L. K. The application of a molecular clock based on molecular sequences and the fossil record to explain biogeographic distributions within the *Alexandrium tamarense* “species complex”(Dinophyceae). *Mol. Phylogenet. Evol.* **20**, 1015-1027 (2003).

14. Tang, X., Yu, R., Zhou, M. & Yu, Z. Application of rRNA probes and fluorescence in situ hybridization for rapid detection of the toxic dinoflagellate *Alexandrium minutum*. *Chin. J. Oceanol. Limnol.* **30**, 256-263 (2012).

15. Logares, R. *et al.* Recent evolutionary diversification of a protist lineage. *Environ. Microbiol.* **10**, 1231-1243 (2008).

16. Kim, K., Yoshida, M. & Kim, C. Molecular phylogeny of three hitherto unreported *Alexandrium* species: *Alexandrium hiranoi*, *Alexandrium leei* and *Alexandrium satoanum* (Gonyaulacales, Dinophyceae) inferred from the 18S and 26S rDNA sequence data. *Phycologia* **44**, 361-368 (2005).

17. Yamada, N., Tanaka, A. & Horiguchi, T. Pigment compositions are linked to the habitat types in dinoflagellates. *J. Plant Res.* **128**, 923-932 (2015).

18. Yamaguchi, A., Hoppenrath, M., Pospelova, V., Horiguchi, T. & Leander, B. S. Molecular phylogeny of the marine sand-dwelling dinoflagellate *Herdmania litoralis* and an emended description of the closely related planktonic genus *Archaeperidinium Jörgensen*. *Eur. J. Phycol.* **46**, 98-112 (2011).

19. Mertens, K. N. *et al.* *Archaeperidinium saanichi* sp. nov.: a new species based on morphological variation of cyst and theca within the *Archaeperidinium minutum* Jörgensen 1912 species complex. *Mar. Micropaleontol.* **96**, 48-62 (2012).

20. Gu, H. & Wang, Y. The First Record of Ensiculifera Balech and Fragilidium Balech (Dinophyceae) from Chinese Coast. *Acta Phytotax. Sin.* **45**, 828-840 (2007).

21. Lin, S., Zhang, H., Hou, Y., Miranda, L. & Bhattacharya, D. Development of a dinoflagellate-oriented PCR primer set leads to detection of picoplanktonic dinoflagellates from Long Island Sound. *Appl. Environ. Microbiol.* **72**, 5626-5630 (2006).

22. Saldarriaga, J. F., Cavalier-Smith, T., Menden-Deuer, S. & Keeling, P. J. Molecular data and the evolutionary history of dinoflagellates. *Eur. J. Protistol.* **40**, 85-111 (2004).

23. Tillmann, U., Gottschling, M., Nézan, E., Krock, B. & Bilien, G. Morphological and molecular characterization of three new *Azadinium* species (Amphidomataceae, Dinophyceae) from the Irminger Sea. *Protist* **165**, 417-444 (2014).

24. Ishitani, Y., Ujiié, Y. & Takishita, K. Uncovering sibling species in Radiolaria: evidence for ecological partitioning in a marine planktonic protist. *Mol. Phylogenet. Evol.* **78**, 215-222 (2014).

25. Ki, J. S. & Han, M. S. Informative characteristics of 12 divergent domains in complete large subunit rDNA sequences from the harmful dinoflagellate genus, *Alexandrium* (Dinophyceae). *J. Eukaryot. Microbiol.* **54**, 210-219 (2007).

26. Qiu, D., Huang, L., Liu, S., Zhang, H. & Lin, S. Apical groove type and molecular phylogeny suggests reclassification of *Cochlodinium geminatum* as *Polykrikos geminatum*. *PloS one* **8**, e71346; 10.1371/journal.pone.0071346 (2013).

27. Potvin, E., Rochon, A. & Lovejoy, C. Cyst–theca relationship of the arctic dinoflagellate cyst *Islandinium minutum* (Dinophyceae) and phylogenetic position based on SSU rDNA and LSU rDNA. *J. Phycol.* **49**, 848-866 (2013).

28. Mertens, K. N. *et al.* A new heterotrophic dinoflagellate from the North‐eastern Pacific, *Protoperidinium fukuyoi*: Cyst–theca relationship, phylogeny, distribution and ecology. *J. Eukaryot. Microbiol.* **60**, 545-563 (2013).

29. Matsuoka, K. & Kawami, H. Phylogenetic subdivision of the genus *Protoperidinium,* (Peridiniales, Dinophyceae) with emphasis on the *Monovela* Group in *Biological and geological perspectives of dinoflagellates* (eds. Marret, F. J. M. & Bradley, L.) 267-276 (The Micropalaeontological Society, Special Publications. Geological Society, 2013).

30. Mertens, K. N. *et al.* Cyst‐theca relationship of a new dinoflagellate with a spiny round brown cyst*, Protoperidinium lewisiae* sp. nov., and its comparison to the cyst of *Oblea acanthocysta*. *Phycol. Res.* **63**, 110-124 (2015).

31. Reñé, A., Camp, J. & Garcés, E. *Polykrikos tanit* sp. nov., a new mixotrophic unarmoured pseudocolonial dinoflagellate from the NW Mediterranean Sea. *Protist* **165**, 81-92 (2014).

32. Kim, S. H. *et al.* Phylogenetic analysis of harmful algal bloom (HAB)-causing dinoflagellates along the Korean coasts, based on SSU rRNA gene. *J. Microbiol. Biotechnol.* **14**, 959-966 (2004).

33. Matsuoka, K., Kawami, H., Nagai, S., Iwataki, M. & Takayama, H. Re-examination of cyst–motile relationships of *Polykrikos kofoidii* Chatton and *Polykrikos schwartzii* Bütschli (Gymnodiniales, Dinophyceae). *Rev. Palaeobot. Palynol.* **154**, 79-90 (2009).

34. Han, M. S. *et al.* Morphological and Molecular Phylogenetic Position of *Prorocentrum micans* *sensu stricto* and Description of *Prorocentrum koreanum* sp. nov. from Southern Coastal Waters in Korea and Japan. *Protist* **167**, 32-50 (2016).

35. Mertens, K. N. *et al.* Process length variation of the cyst of the dinoflagellate *Protoceratium reticulatum* in the North Pacific and Baltic‐Skagerrak region: calibration as an annual density proxy and first evidence of pseudo‐cryptic speciation. *J. Quat. Sci.* **27**, 734-744 (2012).

36. Yamaguchi, A., Kawamura, H. & Horiguchi, T. A further phylogenetic study of the heterotrophic dinoflagellate genus, *Protoperidinium* (Dinophyceae) based on small and large subunit ribosomal RNA gene sequences. *Phycol. Res.* **54**, 317-329 (2006).

37. Ribeiro, S., Lundholm, N., Amorim, A. & Ellegaard, M. *Protoperidinium minutum* (Dinophyceae) from Portugal: cyst–theca relationship and phylogenetic position on the basis of single-cell SSU and LSU rDNA sequencing. *Phycologia* **49**, 48-63 (2010).

38. Gómez, F., Moreira, D. & López-García, P. Molecular phylogeny of noctilucoid dinoflagellates (Noctilucales, Dinophyceae). *Protist* **161**, 466-478 (2010).

39. Takahashi, K., Moestrup, Ø., Jordan, R. W. & Iwataki, M. Two new freshwater woloszynskioids *Asulcocephalium miricentonis* gen. et sp. nov. and *Leiocephalium pseudosanguineum* gen. et sp. nov.(Suessiaceae, Dinophyceae) lacking an apical furrow apparatus. *Protist* **166**, 638-658 (2015).

40. Hoppenrath, M., Bachvaroff, T. R., Handy, S. M., Delwiche, C. F. & Leander, B. S. Molecular phylogeny of ocelloid-bearing dinoflagellates (Warnowiaceae) as inferred from SSU and LSU rDNA sequences. *BMC Evol. Bio.* **9**, 116 (2009).

41. Manrique, J. M., Calvo, A. Y. & Jones, L. R. Phylogenetic analysis of *Ostreococcus* virus sequences from the Patagonian Coast. *Virus genes* **45**, 316-326 (2012).

42. Skovgaard, A., Karpov, S. A. & Guillou, L. The parasitic dinoflagellates *Blastodinium* spp. inhabiting the gut of marine, planktonic copepods: morphology, ecology, and unrecognized species diversity. *Front. Microbiol.* **3**, 305;10.3389/fmicb.2012.00305 (2012).

43. Saldarriaga, J. F., Taylor, F., Keeling, P. J. & Cavalier-Smith, T. Dinoflagellate nuclear SSU rRNA phylogeny suggests multiple plastid losses and replacements. *J. Mol. Evol.* **53**, 204-213 (2001).

44. Gómez, F., Moreira, D. & López-García, P. Life cycle and molecular phylogeny of the dinoflagellates *Chytriodinium* and *Dissodinium*, ectoparasites of copepod eggs. *Eur. J. protistol.* **45**, 260-270 (2009).

45. Coats, D. W., Kim, S., Bachvaroff, T. R., Handy, S. M. & Delwiche, C. F. *Tintinnophagus acutus* n. g., n. sp. (Phylum Dinoflagellata), an ectoparasite of the ciliate *Tintinnopsis cylindrica* Daday 1887, and its relationship to *Duboscquodinium collini* Grassé 1952. *J. Eukaryot. Microbiol.* **57**, 468-482 (2010).

46. Marie, D., Shi, X. L., Rigaut-Jalabert, F. & Vaulot, D. Use of flow cytometric sorting to better assess the diversity of small photosynthetic eukaryotes in the English Channel. *FEMS Microbiol. Ecol.* **72**, 165-178 (2010).

47. Zhang, Q., Liu, G. & Hu, Z. Morphological differences and molecular phylogeny of freshwater blooming species, *Peridiniopsis* spp. (Dinophyceae) from China. *Eur. J. Protistol.* **47**, 149-160 (2011).

48. Kim, S. *et al.* Genetic diversity of parasitic dinoflagellates in the genus *Amoebophrya* and its relationship to parasite biology and biogeography. *J. Eukaryot. Microbiol.* **55**, 1-8 (2008).

49. Reñé, A., Camp, J. & Garcés, E. Diversity and phylogeny of Gymnodiniales (Dinophyceae) from the NW Mediterranean Sea revealed by a morphological and molecular approach. *Protist* **166**, 234-263 (2015).

50. Kretschmann, J., Filipowicz, N. H., Owsianny, P. M., Zinssmeister, C. & Gottschling, M. Taxonomic Clarification of the Unusual Dinophyte *Gymnodinium limneticum* Wołosz.(Gymnodiniaceae) from the Tatra Mountains. *Protist* **166**, 621-637 (2015).

51. Takano, Y., Yamaguchi, H., Inouye, I., Moestrup, Ø. & Horiguchi, T. Phylogeny of five species of *Nusuttodinium* gen. nov.(Dinophyceae), a genus of unarmoured kleptoplastidic dinoflagellates. *Protist* **165**, 759-778 (2014).

52. Aydin, H., Yürür, E. E., Uzar, S. & Küçüksezgin, F. Impact of industrial pollution on recent dinoflagellate cysts in Izmir Bay (Eastern Aegean). *Mar. Pollut. Bull.* **94**, 144-152 (2015).

53. Heikkilä, M. *et al.* Surface sediment dinoflagellate cysts from the Hudson Bay system and their relation to freshwater and nutrient cycling. *Mar. Micropaleontol.* **106**, 79-109 (2014).

54. Satta, C. T. *et al.* Studies on dinoflagellate cyst assemblages in two estuarine Mediterranean bays: A useful tool for the discovery and mapping of harmful algal species. *Harmful Algae* **24**, 65-79 (2013).

55. Sildever, S., Andersen, T. J., Ribeiro, S. & Ellegaard, M. Influence of surface salinity gradient on dinoflagellate cyst community structure, abundance and morphology in the Baltic Sea, Kattegat and Skagerrak. *Estuar. Coast. Shelf Sci.* **155**, 1-7 (2015).

56. Satta, C. T. *et al.* Dinoflagellate cyst assemblages in surface sediments from three shallow Mediterranean lagoons (Sardinia, North Western Mediterranean Sea). *Estuar. Coast.* **37**, 646-663 (2014).

57. Limoges, A., Londeix, L. & de Vernal, A. Organic-walled dinoflagellate cyst distribution in the Gulf of Mexico. *Mar. Micropaleontol.* **102**, 51-68 (2013).

58. D’Silva, M. S., Anil, A. C. & Sawant, S. S. Dinoflagellate cyst assemblages in recent sediments of Visakhapatnam harbour, east coast of India: Influence of environmental characteristics. *Mar. Pollut. Bull.* **66**, 59-72 (2013).

59. Narale, D. D., Patil, J. S. & Anil, A. C. Dinoflagellate cyst distribution in recent sediments along the south-east coast of India. *Oceanologia* **55**, 979-1003 (2013).

60. Narale, D. D. & Anil, A. C. Spatial distribution of dinoflagellates from the tropical coastal waters of the South Andaman, India: Implications for coastal pollution monitoring. *Mar. Pollut. Bull.* **115**, 498-506 (2017).

61. Sildever, S. *et al.* Spring bloom dinoflagellate cyst dynamics in three eastern sub-basins of the Baltic Sea. *Cont. Shelf Res.* **137**, 46-55 (2017).

62. Triki, H. Z. *et al.* Do the levels of industrial pollutants influence the distribution and abundance of dinoflagellate cysts in the recently-deposited sediment of a Mediterranean coastal ecosystem? *Sci. Total Environ.* **595**, 380-392 (2017).

63. Uddandam, P. R., Prasad, V. & Rai, J. Dinoflagellate cyst distribution in sediments of western Bay of Bengal: Role of sea surface conditions. *Palaeogeogr. Palaeoclimatol. Palaeoecol.* **483**, 31-48 (2017).

64. Elshanawany, R. & Zonneveld, K. A. Dinoflagellate cyst distribution in the oligotrophic environments of the Gulf of Aqaba and northern Red Sea. *Mar. Micropaleontol.* **124**, 29-44 (2016).

65. Dhib, A., Fertouna-Bellakhal, M., Turki, S. & Aleya, L. Driving factors of dinoflagellate cyst distribution in surface sediments of a Mediterranean lagoon with limited access to the sea. *Mar. Pollut. Bull.* **112**, 303-312 (2016).

66. Price, A. M., Gurdebeke, P. R., Mertens, K. N. & Pospelova, V. Determining the absolute abundance of dinoflagellate cysts in recent marine sediments III: Identifying the source of *Lycopodium* loss during palynological processing and further testing of the *Lycopodium* marker-grain method. *Rev. Palaeobot. Palynol.* **226**, 78-90 (2016).

67. Aydın, H., Yürür, E. E., Uzar, S. & Küçüksezgin, F. Modern Dinoflagellate Cyst Assemblages of Aliağa and Nemrut Bay: Influence of Industrial Pollution. *Turk. J. Fish. Aquat. Sci.* **15**, 543-554 (2015).

68. Aydın, H., Balcı, M., Uzar, S. & Balkıs, N. Dinoflagellate cyst assemblages in surface sediments of Southwestern Black sea and Çanakkale strait (Dardanelles). *Fresenius Env. Bull.* **12**, 4789-4798 (2015).

69. Aydin, H. & Uzar, S. Distribution and abundance of modern dinoflagellate cysts from Marmara, Aegean and Eastern Seas of Turkey. *J. Environ. Biol.* **35**, 413 (2014).

70. Orlova, T. Y. & Morozova, T. Dinoflagellate cysts in recent marine sediments of the western coast of the Bering Sea. *Russian J. Mar. Biol.* **39**, 15-29 (2013).

71. Richerol, T., Pienitz, R. & Rochon, A. Modern dinoflagellate cyst assemblages in surface sediments of Nunatsiavut fjords (Labrador, Canada). *Mar. Micropaleontol.* **88**, 54-64 (2012).

72. Kim, S. Y., Lim, D. I. & Cho, H. J. Dinoflagellate cyst assemblages from the northern shelf sediments of the East China Sea: An indicator of marine productivity. *Mar. Micropaleontol.* **96**, 75-83 (2012).

73. Candel, M. S., Radi, T., de Vernal, A. & Bujalesky, G. Distribution of dinoflagellate cysts and other aquatic palynomorphs in surface sediments from the Beagle Channel, Southern Argentina. *Mar. Micropaleontol.* **96**, 1-12 (2012).

74. Liu, D. *et al.* The impact of different pollution sources on modern dinoflagellate cysts in Sishili Bay, Yellow Sea, China. *Mar. Micropaleontol.* **84**, 1-13 (2012).

75. Matsuoka, K. & Koike, K. Phytoplankton surveys off the southern Myanmar coast of the Andaman Sea: an emphasis on dinoflagellates including potentially harmful species. *Fish. Sci.* **78**, 1091-1106 (2012).

76. Srivilai, D., Lirdwitayaprasit, T. & Fukuyo, Y. Distribution of dinoflagellate cysts in the surface sediment of the coastal areas in Chonburi Province, Thailand. *Coast. Mar. Sci.* **35**, 11-19 (2012).

77. Rubino, F., Belmonte, M., Caroppo, C. & Giacobbe, M. Dinoflagellate cysts from surface sediments of Syracuse Bay (Western Ionian Sea, Mediterranean). *Deep-Sea Res. PT. II.* **57**, 243-247 (2010).

78. Pospelova, V. & Kim, S. J. Dinoflagellate cysts in recent estuarine sediments from aquaculture sites of southern South Korea. *Mar. Micropaleontol.* **76**, 37-51 (2010).

79. Limoges, A., Kielt, J. F., Radi, T., Ruíz-Fernandez, A. C. & de Vernal, A. Dinoflagellate cyst distribution in surface sediments along the south-western Mexican coast (14.76 N to 24.75 N). *Mar. Micropaleontol.* **76**, 104-123 (2010).

80. Shin, H. H. *et al.* Reconstruction of historical nutrient levels in Korean and Japanese coastal areas based on dinoflagellate cyst assemblages. *Mar. Pollut. Bull.* **60**, 1243-1258 (2010).

81. Satta, C. T. *et al.* Dinoflagellate cysts in recent sediments from two semi-enclosed areas of the Western Mediterranean Sea subject to high human impact. *Deep-Sea Res. PT. II.* **57**, 256-267 (2010).

82. Shin, H. H., Yoon, Y. H., Kim, Y.-O. & Matsuoka, K. Dinoflagellate cysts in surface sediments from southern coast of Korea. *Estuar. Coast.* **34**, 712-725 (2011).

83. Mohamed, Z. A. & Al-Shehri, A. M. Occurrence and germination of dinoflagellate cysts in surface sediments from the Red Sea off the coasts of Saudi Arabia. *Oceanologia* **53**, 121-136 (2011).

84. Krepakevich, A. & Pospelova, V. Tracing the influence of sewage discharge on coastal bays of Southern Vancouver Island (BC, Canada) using sedimentary records of phytoplankton. *Cont. Shelf Res.* **30**, 1924-1940 (2010).

85. Elshanawany, R., Zonneveld, K., Ibrahim, M. I. & Kholeif, S. E. Distribution patterns of recent organic-walled dinoflagellate cysts in relation to environmental parameters in the Mediterranean Sea. *Palynol.* **34**, 233-260 (2010).
